# Phenotypes of CF rabbits generated by CRISPR/Cas9-mediated disruption of the CFTR gene

**DOI:** 10.1172/jci.insight.139813

**Published:** 2021-01-11

**Authors:** Jie Xu, Alessandra Livraghi-Butrico, Xia Hou, Carthic Rajagopalan, Jifeng Zhang, Jun Song, Hong Jiang, Hong-Guang Wei, Hui Wang, Mohamad Bouhamdan, Jinxue Ruan, Dongshan Yang, Yining Qiu, Youming Xie, Ronald Barrett, Sharon McClellan, Hongmei Mou, Qingtian Wu, Xuequn Chen, Troy D. Rogers, Kristen J. Wilkinson, Rodney C. Gilmore, Charles R. Esther, Khalequz Zaman, Xiubin Liang, Michael Sobolic, Linda Hazlett, Kezhong Zhang, Raymond A. Frizzell, Martina Gentzsch, Wanda K. O’Neal, Barbara R. Grubb, Y. Eugene Chen, Richard C. Boucher, Fei Sun

**Affiliations:** 1 Center for Advanced Models for Translational Sciences and Therapeutics, University of Michigan (UM) Medical Center, Ann Arbor, Michigan, USA.; 2 Marsico Lung Institute, University of North Carolina School of Medicine, Chapel Hill, North Carolina, USA.; 3 Department of Physiology,; 4 Department of Oncology, Karmanos Cancer Institute,; 5 Center for Molecular Medicine and Genetics, and; 6 Department of Anatomy and Cell Biology, Wayne State University (WSU) School of Medicine, Detroit, Michigan, USA.; 7 Mucosal Immunology & Biology Research Center, Massachusetts General Hospital, Boston, Massachusetts, USA.; 8 Department of Pediatrics, Case Western Research University School of Medicine, Cleveland, Ohio, USA.; 9 Department of Pediatrics and Cell Biology, University of Pittsburgh, Pittsburgh, Pennsylvnia, USA.

**Keywords:** Cell Biology, Pulmonology, Genetic diseases, Ion channels

## Abstract

Existing animal models of cystic fibrosis (CF) have provided key insights into CF pathogenesis but have been limited by short lifespans, absence of key phenotypes, and/or high maintenance costs. Here, we report the CRISPR/Cas9-mediated generation of CF rabbits, a model with a relatively long lifespan and affordable maintenance and care costs. CF rabbits supplemented solely with oral osmotic laxative had a median survival of approximately 40 days and died of gastrointestinal disease, but therapeutic regimens directed toward restoring gastrointestinal transit extended median survival to approximately 80 days. Surrogate markers of exocrine pancreas disorders were found in CF rabbits with declining health. CFTR expression patterns in WT rabbit airways mimicked humans, with widespread distribution in nasal respiratory and olfactory epithelia, as well as proximal and distal lower airways. CF rabbits exhibited human CF–like abnormalities in the bioelectric properties of the nasal and tracheal epithelia. No spontaneous respiratory disease was detected in young CF rabbits. However, abnormal phenotypes were observed in surviving 1-year-old CF rabbits as compared with WT littermates, and these were especially evident in the nasal respiratory and olfactory epithelium. The CF rabbit model may serve as a useful tool for understanding gut and lung CF pathogenesis and for the practical development of CF therapeutics.

## Introduction

Mutations of the CFTR gene lead to cystic fibrosis (CF), one of the most common life-threating autosomal recessive disorders (1). The disease is complex because it affects multiple organs, with lung disease producing most CF morbidity and mortality (1–5). There has been major progress in understanding CF pathogenesis since the cloning of the CFTR gene in 1989 (6). However, many important questions remain unanswered related to disease pathogenesis in numerous organs and organ-level responsiveness to therapy (1, 7).

Animal models of CF have undoubtedly contributed to the understanding of disease pathogenesis and the development of therapeutic reagents (8), yet the existing models (mice, rats, ferrets, sheep, and pig) have limitations. From a scientific perspective, they fail to either reproduce major pulmonary phenotypes observed in CF patients or resolve key pathophysiologic questions (e.g., the relative roles of abnormal airway superficial epithelium versus submucosal glands [SMG] in the pathogenesis of CF lung disease; refs. 9–14). From a practical perspective, the larger animal models either live for only short times, impose high maintenance costs, and/or require special animal care, which limits their availability for evaluation of therapeutic agents (8, 10, 15).

In this study, we report the generation and characterization of CF rabbit models. The rabbit was selected as a laboratory-friendly species (16) with features suitable to address CF disease–specific questions. With respect to molecular pathogenesis/therapy, the amino acid sequence of rabbit CFTR exhibits an ~92% identity to human CFTR, which is one of the highest among the animals utilized to generate CF animal models (Supplemental Table 1; supplemental material available online with this article; https://doi.org/10.1172/jci.insight.139813DS1). With respect to disease pathogenesis, the rabbit lung has no SMG and has an architecture resembling human distal airways (16–19), allowing study of the distal airway region in CF lung disease pathogenesis (20, 21). Our study focused on the phenotypic characterization of CF rabbits generated by the CRISPR/Cas9 technology, with emphasis on general survival/nutritional aspects of the model, characterization of rabbit CFTR expression and function, and organ-specific phenotypes.

## Results

### Generation of CF rabbits using CRISPR/Cas9 technology.

CF founder rabbits were produced by CRISPR/Cas9 using a well-established gene-editing platform in New Zealand White (NZW) rabbits (22–24). Due to the inherent nature of CRISPR/Cas9-mediated gene targeting in which the specific mutation types are generally unpredictable, founder animals carried different mutation alleles. Founder animals were bred with WT rabbits to verify germline transmission, and 3 lines of CF rabbits were established carrying the following insertion or deletion (indel) types: (a) CFΔ1, with 1 nucleotide deletion (Δ1) that generates a premature stop codon after amino acid 477; (b) CF+1, with 1 nucleotide insertion (+1) that generates a premature stop codon after amino acid 480; and (c) CFΔ9, with a 9-nucleotide deletion (Δ9) that results in deletion of amino acids P477, S478, and E479 (Supplemental Figure 1, A–C). No off-target mutations were detected in the animals tested (Supplemental Table 2 and 3). Rabbit lines were expanded and characterized at 3 different sites (i.e., WSU [lines CFΔ1, CFΔ9, and CF+1], UM [lines CFΔ1, CFΔ9, and CF+1], and University of North Carolina at Chapel Hill [UNC, line CFΔ1]).

### Breeding and fertility.

Initial characterization of genotype frequency was conducted by breeding 28 CFΔ1 heterozygous (Het) littermates at WSU. This cross produced 186 kits with a genotype distribution of 48 CFTR^+/+^ (WT), 96 CFTR^+/–^ (Het), and 42 CFTR^–/–^ (CF) animals (Supplemental Figure 1D), a near-Mendelian ratio of 1:2:1. Similar distributions of genotypes were observed in CF+1 and CFΔ9 litters (not shown).

Male CFΔ1 CF rabbits crossed with Het females proved to be infertile (*n* = 4). Examination of two CFΔ1 CF males revealed complete atrophy/absence of the vas deferens and epididymis (Supplemental Figure 2), a phenotype common in male people with CF (PwCF) (25) and also observed in CF pigs (26), sheep (10), ferrets (27), and rats (11). In contrast, preliminary studies suggest that CF female rabbits are fertile (*n* = 2).

### Survival, growth curves, and functional expression of CFTR in the rabbit gastrointestinal tract.

**Figure 1:**
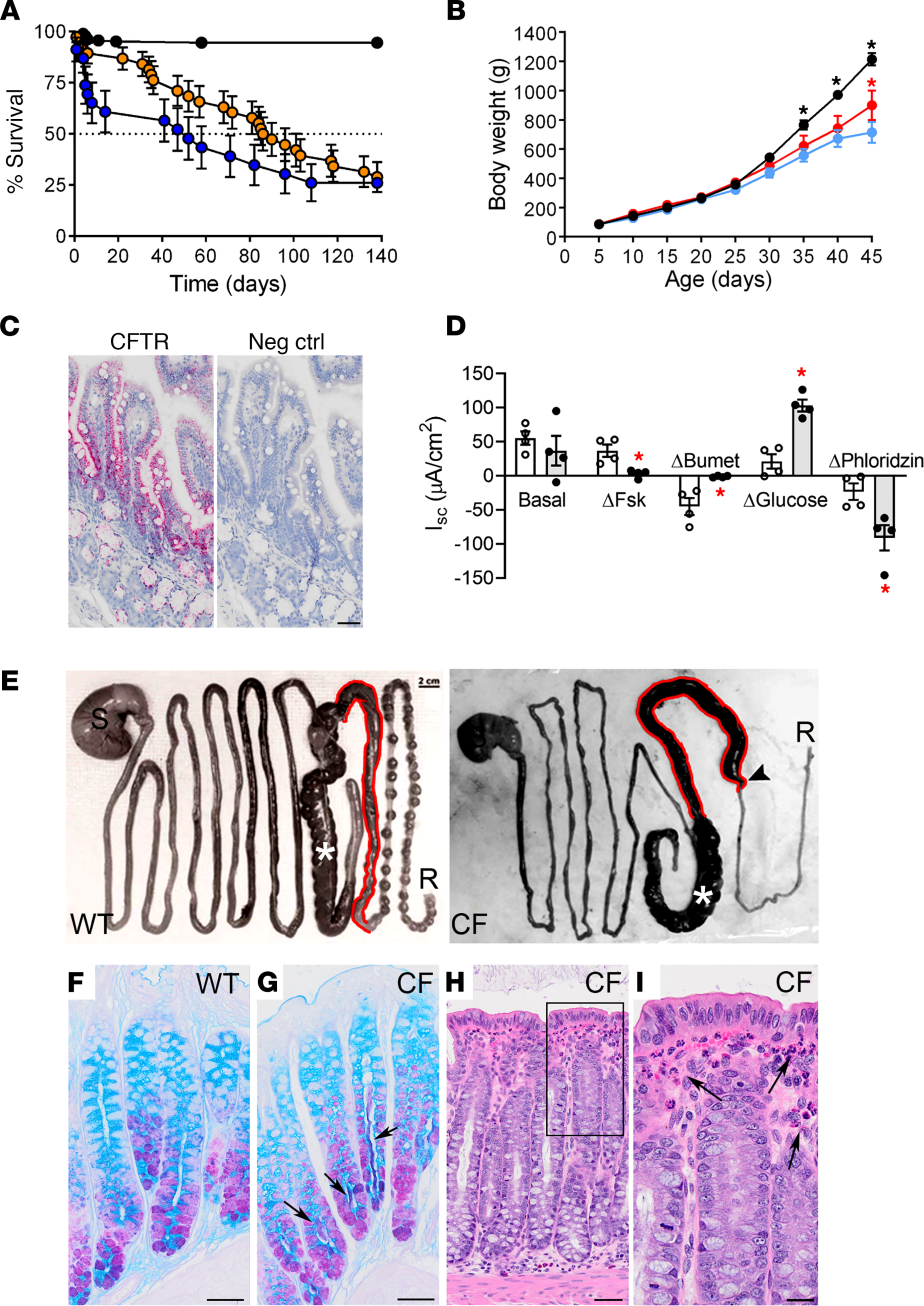
Characterization of CF rabbit gastrointestinal function, weight gain, and mortality. (**A**) Survival curves for WT and heterozygous rabbits (black, *n* = 190), CF rabbits on Golytely (blue, *n* = 23), and CF rabbits on Golytely, Bio-Serve, and abdominal palpation-directed GI therapy (orange, *n* = 38). (**B**) Weight gain for WT rabbits (black, *n* = 32), untreated CF rabbits (blue, *n* = 17), and CF rabbits treated with Golytely starting at P14 (red, *n* = 15). *P < 0.05 versus untreated CF, 2-way ANOVA with Tukey’s test for multiple comparisons. (**C**) mRNA in situ hybridization for rabbit CFTR or negative control (RNAscope, red chromogen) on sections of WT jejunum. Scale bar: 50 μm. Representative micrographs from *n* = 2 rabbits. (**D**) Ussing chamber characterization of freshly excised jejunal tissue from WT (white bar, white dots) and CF (gray bar, black dots) rabbits at approximately P52, illustrating short-circuit currents (I_sc_) under basal conditions, and after forskolin stimulation (ΔFsk), bumetanide inhibition (ΔBumet), glucose addition (Δglucose), and phloridzin inhibition. *n* = 4/genotype, *P < 0.05 versus WT. Unpaired, 2-tailed *t* test with Welch’s correction for unequal variance. (**E**) Freshly excised WT and CF gastrointestinal tract, deconvoluted from stomach (S) to rectum (R). Asterisk represents cecum. The proximal colon (highlighted in red) was dilated in CF rabbits as compared with WT, and there was a paucity of stool pellets in the distal colon of CF rabbits. Arrow indicates a site of obstruction. (**F** and **G**) (AB-PAS stained cross section of distal colon in approximately 3 months-old WT (**F**) (Scale bar: 0.1 mm) and CF (**G**) (Scale bar: 0.1mm) rabbits, demonstrating mucus retention (arrows) in the crypts of CF rabbits. (**H** and **I**) H&E-stained cross section of distal colon in approximately 3 months-old CF rabbit (**H**) (Scale bar: 50 μm) and high magnification of region of interest (**I**) (Scale bar: 20 μm) illustrating granulocytic infiltrates (arrows) in the intestinal submucosa. Representative micrographs from *n* = 4 CF and 4 WT rabbits.

NZW rabbits have a lifespan (7–10 years) that is comparable with both pigs and ferrets (28). Survival curves of CF rabbits, exemplified by the CFΔ1 line, showed that CF rabbits spontaneously drinking an osmotic laxative (Golytely) starting at P14 had a median survival age (i.e., t_1/2_) of 44 days, significantly lower than their WT littermates (Figure 1A). Intestinal stasis/obstruction could be detected in CF rabbits by routine abdominal palpation as early as P14, and a protocol was developed to administer a cocktail of mucokinetic agents when intestinal masses were detected. This cocktail had an effectiveness of about 70% to eliminate palpable masses and restore weight gain in weaned CF rabbits. Rabbits treated with this regimen had a median age of survival > 80 days (Figure 1A), suggesting that medical therapy can alleviate intestinal disease in CF rabbits and extend their lifespan as compared with other large CF animal models, where surgical or transgenic correction of the CFTR defect is required to overcome early mortality (10, 27, 29).

We next assessed the growth kinetics of CF versus WT rabbits. At birth, CF kits exhibited no significant differences in body weight or appearance compared with WT littermates. However, after P30, surviving CF kits failed to gain weight at the same rate as their WT counterparts (Figure 1B, CFΔ1 line) and exhibited smaller size (Supplemental Figure 3A), a feature typical of CF children (30). Early administration of Golytely significantly improved, but did not fully correct, the weight gain of CF rabbits (Figure 1B).

CFTR is widely expressed in the rabbit gastrointestinal (GI) tract, including jejunum and colon (Figure 1C and Supplemental Figure 3B, respectively), as detected by mRNA in situ hybridization (ISH). Accordingly, we tested whether CF rabbits recapitulated human CF–like abnormalities in intestinal ion transport. Typical of CF jejunum in humans and in other CF animal models, forskolin-stimulated (Fsk-stimulated; MilliporeSigma) and bumetanide-inhibitable anion secretion (MilliporeSigma) were absent in CF rabbit jejunum at both younger and older ages (mean P52 [Figure 1D] and P367 [Supplemental Figure 3C]), consistent with loss of CFTR function (11, 31–36). Of note, an increase in Na^+^-dependent glucose transport (i.e., phloridzin-sensitive short-circuit currents [I_sc_]; MilliporeSigma) was observed in the jejunum of young CF rabbits (Figure 1D). We speculate that this phenotype was a response to abnormal nutrition (37, 38), as it was not observed in clinically stable, older CF rabbits (Supplemental Figure 3C).

### Intestinal disease in CF rabbits.

**Figure 2:**
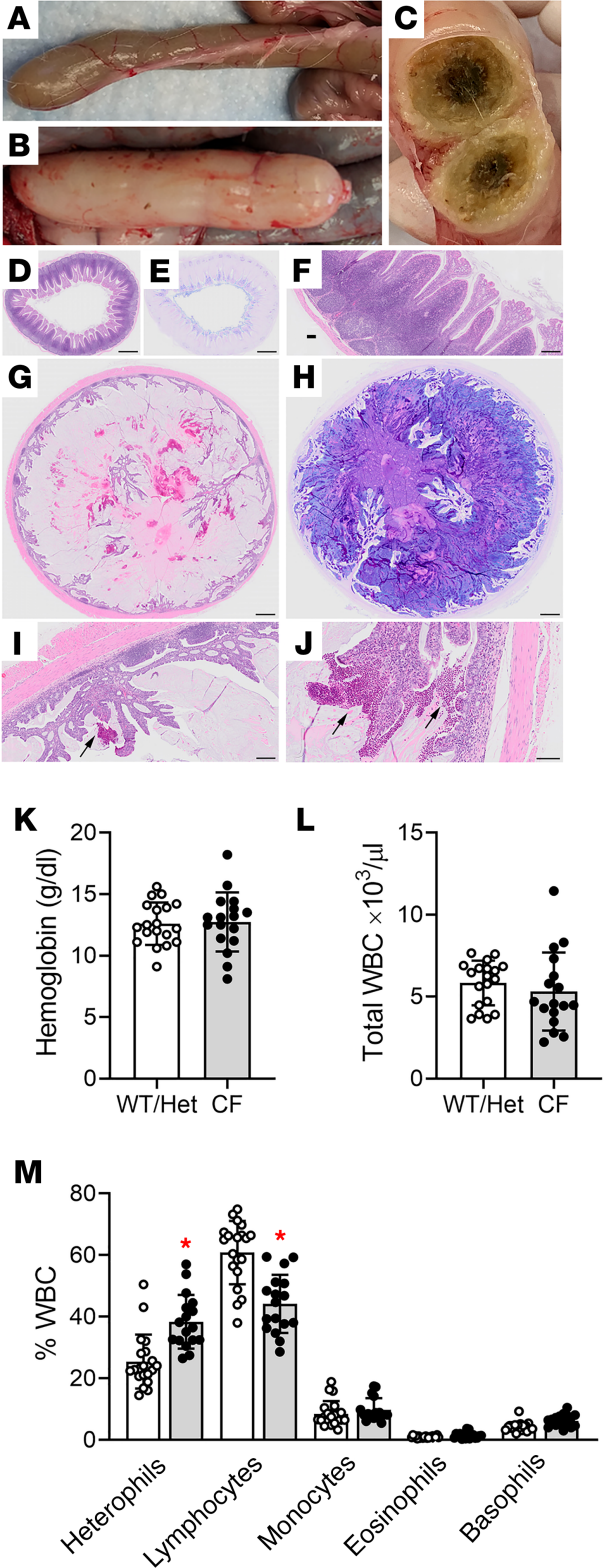
Evidence of systemic inflammation in CF rabbits. (**A**–**C**) Representative gross appearance of the vermiform appendix isolated from approximately 1-year-old WT (**A**) and CF (**B**) rabbits. CF rabbits exhibited swollen and discolored appendix as compared with WT counterparts. When cut in cross section (**C**), the CF appendix lumen was impacted with solid caseous material and remnants of cecal content. (**D** and **E**) Representative histology micrographs of the vermiform appendix isolated from approximately 1-year-old WT rabbit. Cross section, H&E (**D**) and AB-PAS (**E**) stain. Scale bar: 1 mm. (**F**) Higher magnification of the appendix mucosa in WT rabbits, H&E stain. Scale bar: 0.2 mm. (**G** and **H**) Representative histology of the vermiform appendix isolated from approximately 1-year-old CF rabbit, illustrating marked enlargement (**G**, H&E stain) and impaction with AB-PAS^+^ material (**H**, AB-PAS stain). Scale bar: 1 mm. (**I** and **J**) Higher magnification of the CF appendix exhibiting grossly simplified epithelial structures compared with WT, barely detectable lymphoid tissue, and intraluminal accumulation of granulocytes (arrows). H&E stain. Scale bar: 0.2 mm (**I**) and 0.1 mm (**J**). Representative micrographs from *n* = 5 CF and WT rabbits. (**K**–**M**) Hematologic values for WT and CF rabbits at approximately 4–5 months of age. Total hemoglobin (**K**), white blood cell (WBC) total (**L**), and differential cell counts (**M**). *n* = 19 WT (white bars, white dots) and 17 CF (gray bars, black dots) rabbits. *P < 0.05 versus WT. Unpaired, 2-tailed *t* test.

We next investigated the mode of death of CF kits. In humans, one of the earliest manifestations of CF is meconium ileus (MI), a small intestine obstruction that occurs in approximately 15%–20% of newborns with CF (39–42). MI at variable penetrance occurs in all previously described CF animal models within the first few days after birth (10, 27, 29). Although CF rabbits did not exhibit perinatal MI (even if not administered osmotic laxative), intestinal dysfunction characterized by obstruction of the proximal colon developed before weaning (Figure 1E). When not resolved by pharmacological intervention, this intestinal obstruction led to death. Alcian blue–periodic acid Schiff–stained (AB-PAS–stained) cross sections of distal colon demonstrated mucus stasis in the colonic crypts that selectively occurred in CF rabbits, even during administration of Golytely (Figure 1, F and G). Histopathology of CF rabbit colon also revealed submucosal infiltration of inflammatory cells, including heterophils (i.e., the rabbit equivalent of neutrophils) (43) (Figure 1, H and I). Moreover, in young (<2-month-old) rabbits, the vermiform appendix appeared normal but became routinely dilated with atrophic epithelium and intraluminal accumulation of solid caseous material and inflammatory cells in older (>4-month-old) CF rabbits as compared with WT or Het controls (Figure 2, A–J).

GI disease can contribute to hematologic abnormalities in CF children due to malabsorption of nutrients (44, 45). Accordingly, routine analyses of blood counts were performed on 4- to 5-month-old WT and CF rabbits. Hemoglobin and total white blood cell (WBC) counts were similar in CF versus WT rabbits (Figure 2, K and L). However, the WBC differential counts revealed increased percent heterophils and decreased percent lymphocytes in CF rabbits (Figure 2M), consistent with an ongoing inflammatory process. Alternatively, neutrophilia associated with lymphopenia could be due to a differential response to stress in CF versus WT rabbits (stress leukogram).

**Figure 3:**
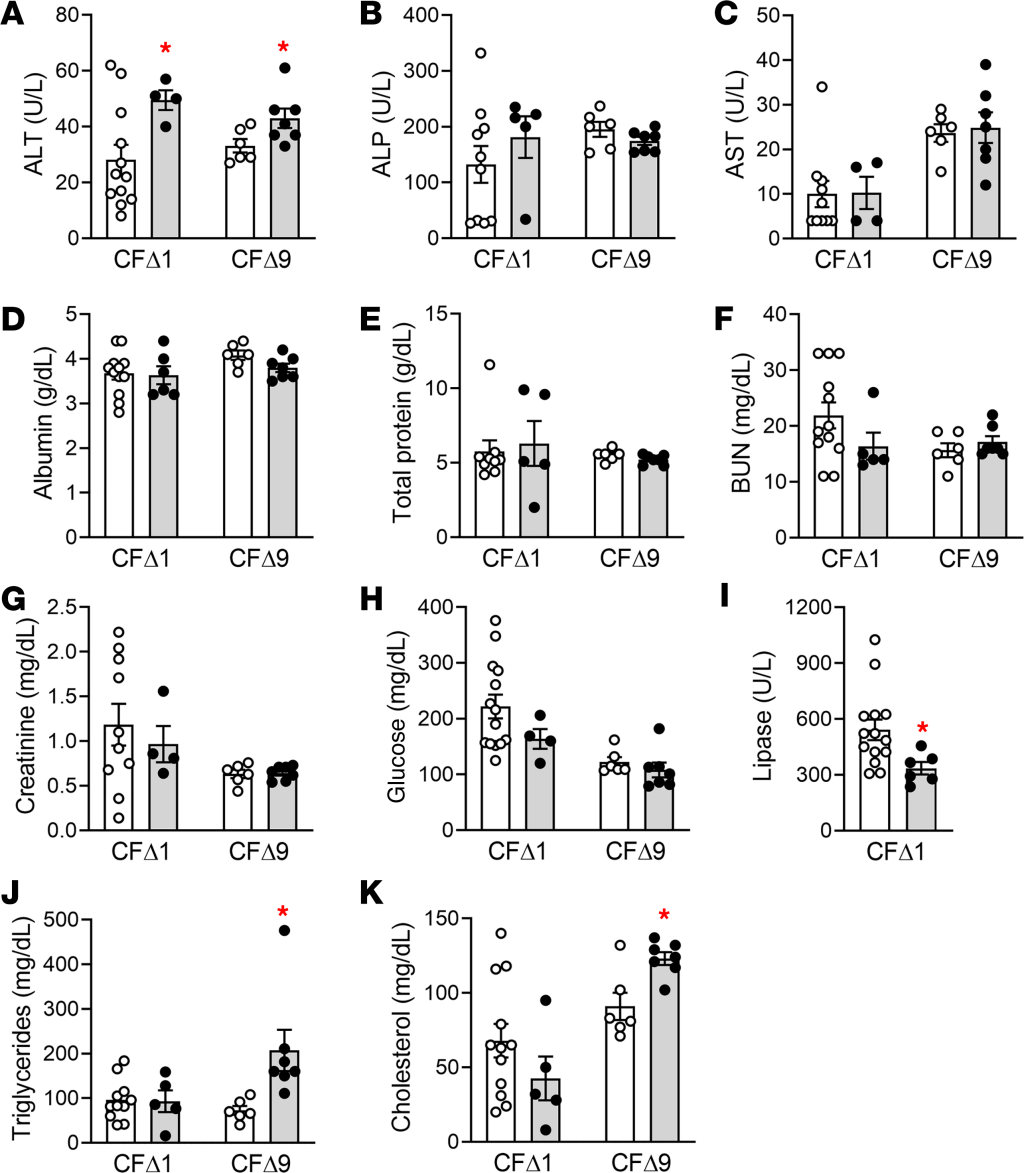
Blood metabolic panel in WT and CF rabbits. (**A**–**K**) Blood chemistry panel for liver, pancreatic, and renal parameters in 2- to 3-month-old WT and CF rabbits from the Δ1 line raised at UNC (*n* = 6–14 WT/Het, white column, white dots; *n* = 4–6 CF, gray column, black dots) or from the Δ9 line raised at UM (*n* = 6 WT white column, white dots; *n* = 7 CF, gray column, black dots). All rabbits were in stable clinical conditions at collection. ALT, Alanine aminotransferase; ALP, alkaline phosphatase; AST, aspartate aminotransferase; BUN, blood urea nitrogen. *P < 0.05 CF versus WT, unpaired, 2-tailed *t* test.

A metabolic panel measuring hepatic, pancreatic, intestinal, and renal function was obtained for clinically stable, 2- to 3-month-old rabbits from the CFΔ1 line raised at UNC and for the CFΔ9 line raised at UM (Figure 3, A–K). A similar panel was also obtained for clinically stable, 1-year-old rabbits from the CFΔ1 line raised at UNC (Supplemental Figure 4, A–K). Overall, the measured values for all analytes fell within normal ranges for NZW rabbits (43). However, few analytes were significantly different between CF versus WT controls. In particular, aminotransferase (ALT) levels were slightly higher in CF than WT rabbits in both cohorts, which could indicate liver abnormalities (43) (Figure 3A). To probe for liver pathology in situ, collagen deposition around the portal triads was evaluated as a hallmark of the biliary cirrhosis and bile duct hyperplasia observed in PwCF (3), but liver abnormalities were not detected in either cohort (Supplemental Figure 4L). Measurements of rabbit plasma trypsinogen were not technically feasible. Even if not always of the same direction between the 2 lines, abnormalities in lipid metabolism were observed for both Δ1 and Δ9 lines and involved plasma lipase, triglycerides, and cholesterol. Reduced levels of plasma lipase (measured at UNC in both age cohorts and significantly different in the younger one; Figure 3I and Supplemental Figure 4I) are consistent with pancreatic acinar dysfunction. Altered triglycerides (Figure 3J) and cholesterol (Figure 3K) levels observed in CF rabbits could reflect, in part, reduced intestinal lipase activity, as well as contributions from reduced bile acid secretion and gut wall abnormalities.

Unlike in mice, CFTR mRNA was easily detectable by mRNA ISH in rabbit pancreas, with a distribution consistent with expression in intercalated ducts/centriacinar cells (Supplemental Figure 5A). Histological evaluation of pancreatic tissues collected from the UM CFΔ9 cohort revealed foci of exocrine gland distension, fibrosis, and inflammatory cell infiltration (3 of 5 CF rabbits; Supplemental Figure 5B), whereas healthy Δ1 CF rabbits in the UNC cohorts revealed no significant difference in the morphology of exocrine or endocrine components compared with WT littermates (Supplemental Figure 5C).

### Loss of function and structural abnormalities in CF rabbit airways.

CFTR expression and function in the airways is species specific (46). Accordingly, the upper and lower airways of WT and CF rabbits were probed with a variety of methods to test for CFTR mRNA transcript, protein expression, and function.

**Figure 4:**
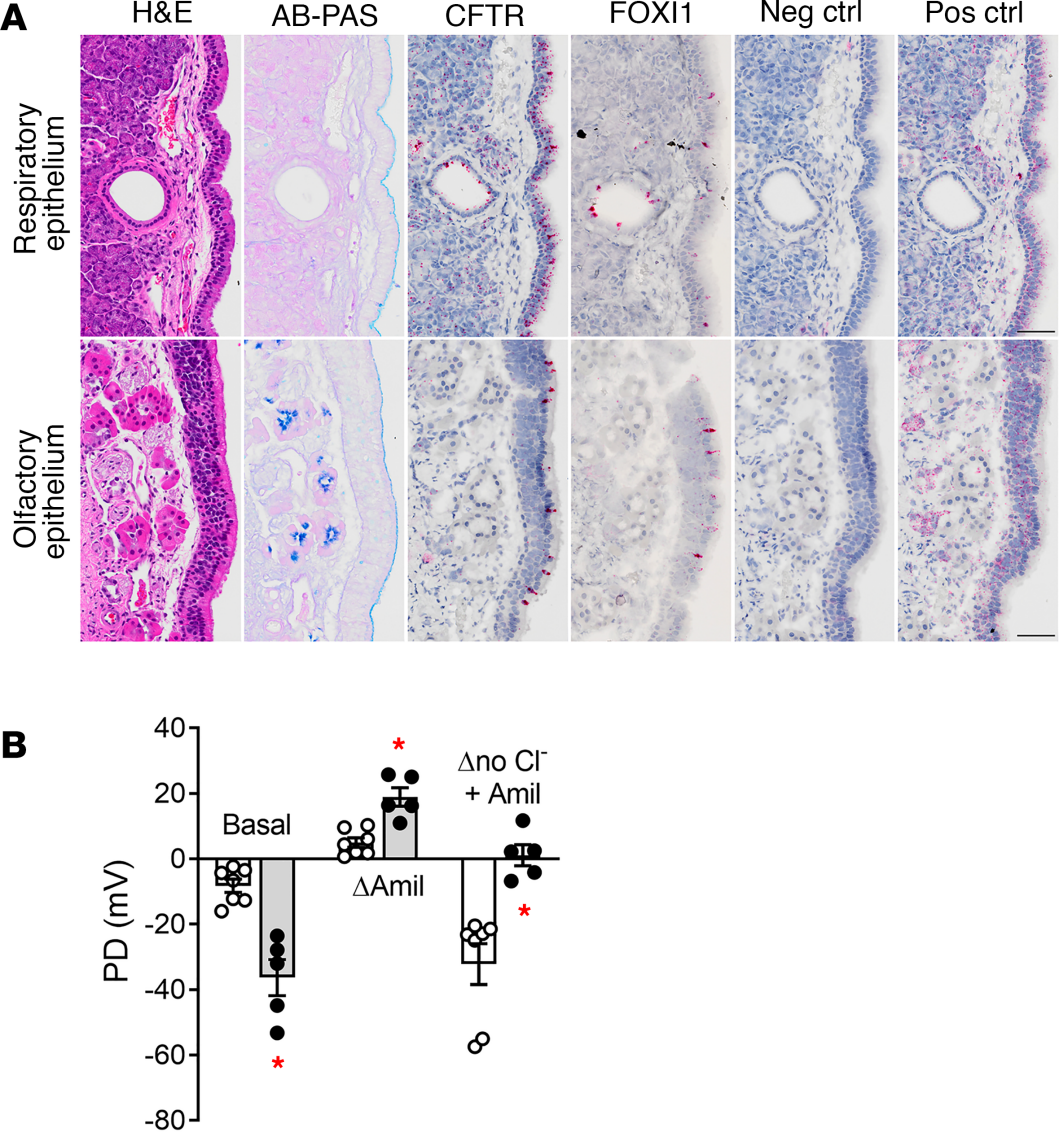
Characterization of CFTR expression and function in the rabbit nasal cavity. (**A**) Representative micrographs of respiratory and olfactory epithelium from WT rabbits (approximately 3-month-old), stained with H&E and AB-PAS, and probed for CFTR, FOXI1, negative control (bacterial gene 4-hydroxy-tetrahydrodipicolinate reductase), and positive control (rabbit gene peptidylprolyl isomerase) mRNA by RNAscope (red chromogen). Scale bar: 50μm. Representative micrographs from *n* = 2 rabbits. (**B**) In vivo measurements of transepithelial nasal potential difference (NPD) in WT (white bar, white dots) and CF (gray bar, black dots) rabbits. Basal, amiloride sensitive, and Cl^–^ free with amiloride (no Cl^–^ + amil) PD responses measured in the left nostril are shown. Equivalent measurements were obtained for the right nostril. *n* = 7 WT and 5 CF rabbits. *P < 0.05 versus WT. Unpaired, 2-tailed *t* test with Welch’s correction for unequal variance.

In the WT rabbit nose, the distribution of rabbit CFTR mRNA in the nasal respiratory epithelium was widespread in the superficial epithelium, a pattern not consistent with a low-incidence, ionocyte-centered expression pattern (47, 48), as defined by focal FOXI1 expression (Figure 4A, upper panels). In contrast, rabbit CFTR was clustered in isolated cells in the olfactory epithelium, with a pattern similar to FOXI1^+^ cells (Figure 4A, lower panels). In comparison, mouse CFTR expression was highly clustered in both the respiratory and olfactory nasal epithelia (Supplemental Figure 6).

The airway bioelectric hallmark of human CF is the abnormal nasal potential difference (NPD) responses to sequential perfusion with Krebs-Ringer solution (basal), amiloride (Na^+^ channel blocker), and Cl^–^-free solutions (49). CF rabbits exhibited increased basal and amiloride-sensitive NPD responses, and no Cl^–^-free NPD responses compared with WT (exemplified in CFΔ1 line, Figure 4B), a phenotype virtually identical to human CF subjects. Ion fluxes measured in freshly excised human CF nasal epithelia have documented that the CF bioelectric abnormalities correlate with raised Na^+^ absorption and an absence of CFTR-mediated Cl^–^ (anion) secretion (50, 51). As shown in Figure 4B, the amiloride-sensitive NPD response was greater in CF than WT rabbits, consistent with accelerated Na^+^ absorption. However, given the interactions between Na^+^ and Cl^–^ conductances in determining the magnitude of amiloride responses (50, 52), ion fluxes will be required to confirm whether Na^+^ absorption is increased in CF rabbit noses.

**Figure 5:**
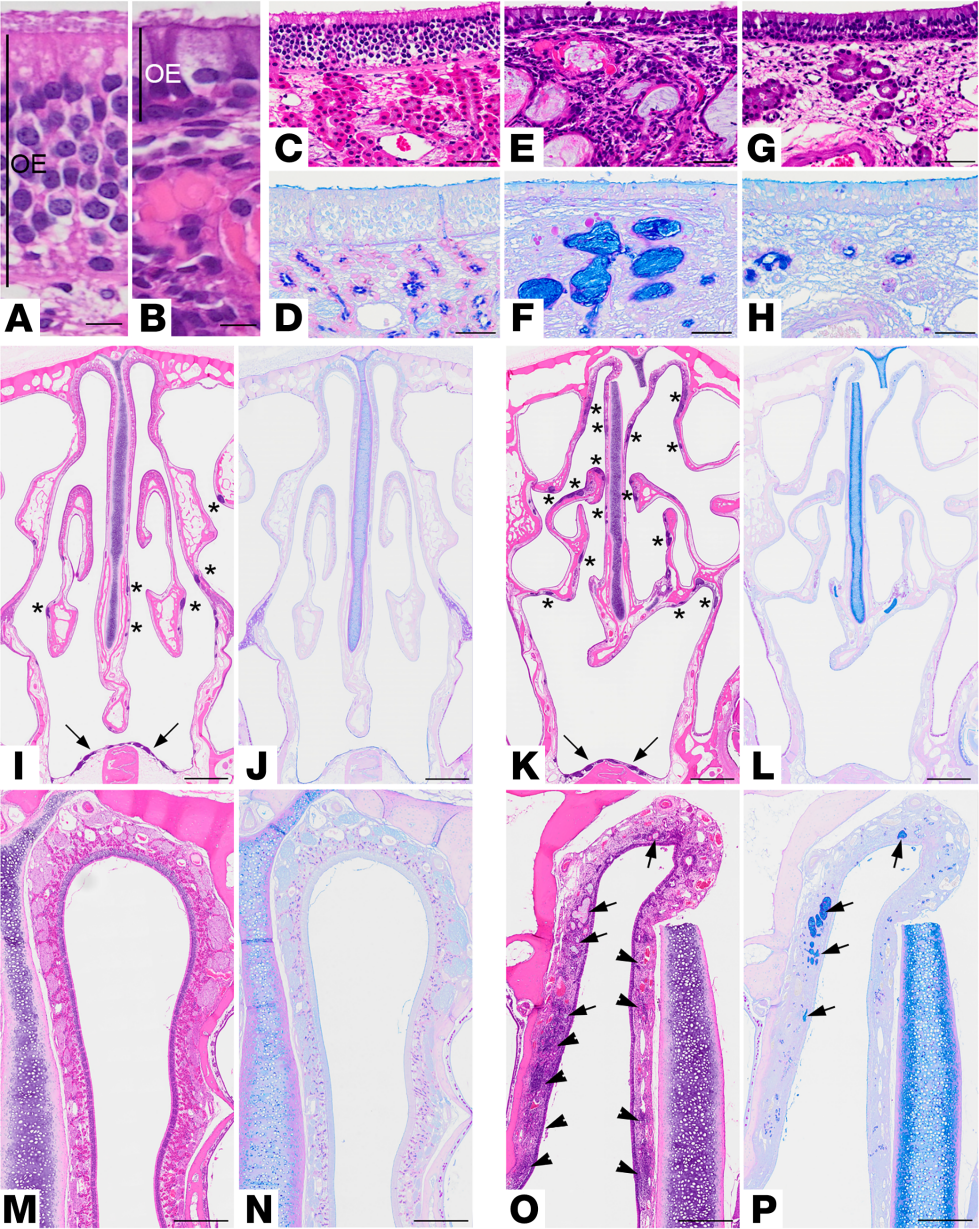
Functional deletion of CFTR in rabbit nose causes progressive remodeling of the olfactory and respiratory mucosa. (**A**–**H**) Representative high-power histological micrographs of WT (**A**, **C**, **D**) and CF (**B**, and **E**–**H**) olfactory mucosa stained with (H&E) (**A**, **B**, **C**, **E**, **G**) or AB-PAS (**D**, **F**, **H**). Scale bar: 10 μm (**A** and **B**), 50 μm (**C**–**H**). Note the striking degeneration of the olfactory epithelium in CF (**B**), which is reduced in height by approximately half, as compared with WT rabbits (**A**). OE, olfactory epithelium. Only sustentacular cells appear to remain on the CF surface epithelium. Degeneration of the olfactory epithelium is accompanied by impaction (**E** and **F**) and atrophy (**G** and **H**) of the underlying Bowman glands. Micrographs representative of *n* = 5 CF and WT rabbits, all ≥ 1 year old. (**I**–**P**) Lower magnification of histological micrographs of WT (**I**, **J**, **M**, and **N**) and CF (**K**, **L**, **O**, and **P**) rabbit posterior nasal cavity stained with H&E (**I**, **K**, **M**, and **O**) or AB-PAS (**J**, **L**, **N**, and **P**). Scale bar: 2 mm (**I**–**L**) and 0.5mm (**M**–**P**). **I**–**L** illustrate the increased number of ectopic lymphocytic aggregates (asterisks) in CF as compared with WT rabbits. Nasal-associated lymphoid tissue (NALT, arrows) was visible in the ventral aspect and do not appear different in the 2 genotypes. **M**–**P** highlight the extent of pathological changes in the nasal cavity of CF rabbit ≥ 1 year old, including remodeling of submucosal glands underlying the olfactory epithelia (**O** and **P**, arrows) and lymphocytic inflammation in the airway epithelial submucosa (**O**, arrowheads).

Morphologically, CF rabbits surviving more than 9 months exhibited degeneration of the olfactory epithelium compared with WT littermates (*n* = 4 of 5; Figure 5, A and B). This phenotype was accompanied by marked remodeling (i.e., atrophy, distension, and mucus impaction) of the Bowman glands in the submucosa underlying the olfactory epithelium (Figure 5, C–H). These phenotypes were not observed in younger rabbits (*n* = 3, 2–4 months old). Both olfactory epithelial degeneration and SMG remodeling have also been observed in CF mice (53) and pigs (54), and an increased abundance of AB-PAS^+^ muco-secretory cells in the nasal respiratory epithelium has been reported for CF rats (11). Seemingly unique to the CF rabbit model, submucosal inflammation in the form of ectopic lymphocytic aggregates or diffuse infiltrates was a consistent observation throughout the nasal cavity of CF rabbits older than 9 months, affecting both the respiratory and olfactory submucosa (Figure 5, I–P, and Supplemental Figure 7).

**Figure 6:**
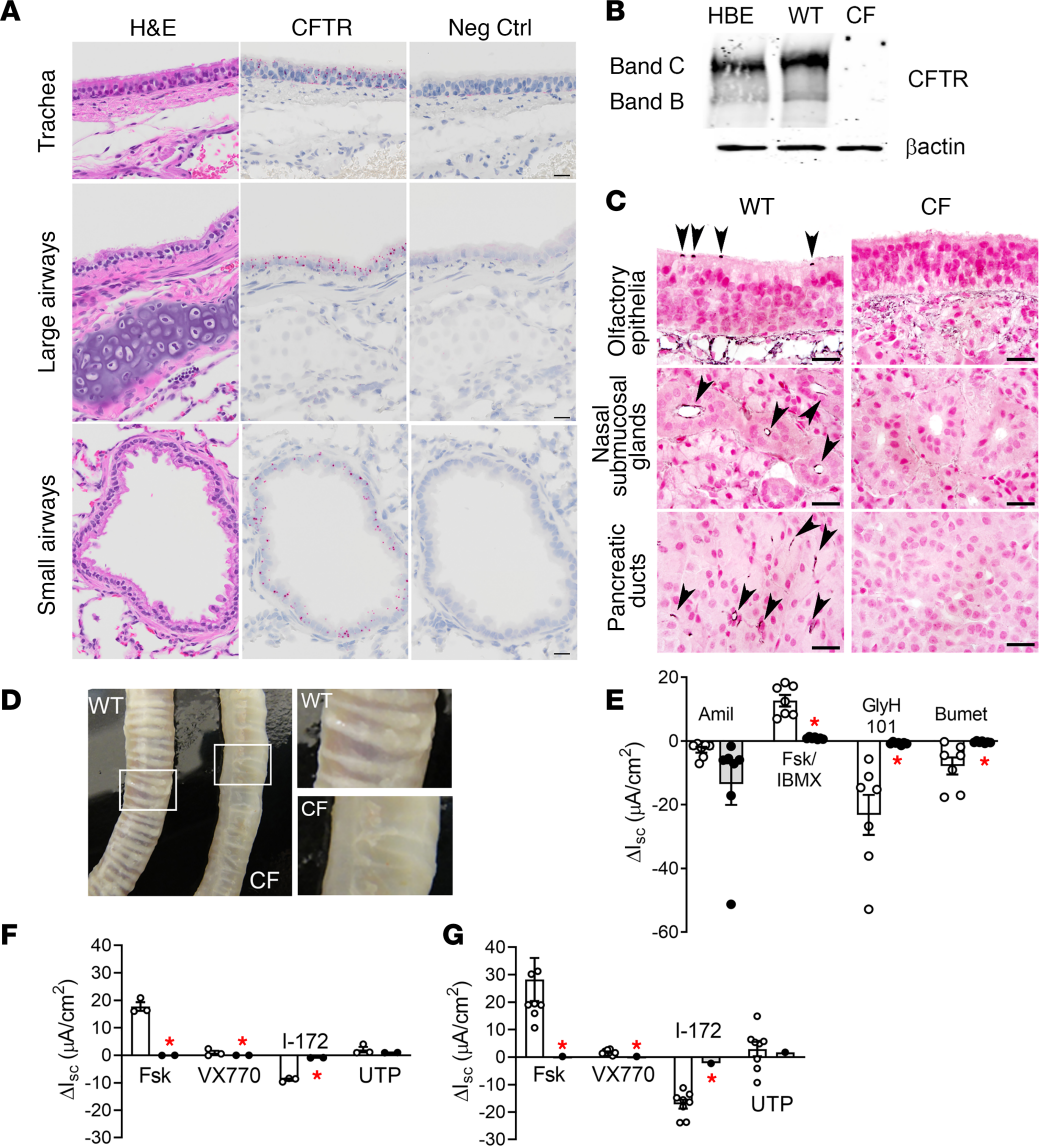
Characterization of rabbit CFTR expression and function in the lower airways. (**A**) Representative micrographs of trachea, large bronchi, and small airways (bronchiole) from WT rabbits (approximately 3 months old), stained with H&E or probed for CFTR and negative control mRNA by RNAscope (red chromogen). Scale bar: 20 μm. *n* = 2 rabbits. (**B**) CFTR Western blots for normal human bronchial epithelial (HBE) cell cultures alongside tracheal tissue lysates from WT and CF rabbits (Δ1 line). Position of band B and C are indicated, and Western blot for β actin is shown as loading control. (**C**) IHC for CFTR in nasal olfactory epithelia, nasal submucosal glands, and pancreas, where CFTR (black chromogen, arrowheads) was detected in cells consistent with the distribution of ionocytes, ductal cells, and collecting ducts, respectively. Scale bar: 20 μm. (**D**) Gross images of tracheas isolated from WT and CF rabbits. CF tracheas lack concentric and equally spaced cartilaginous rings compared with WT rabbits. (**E**) Ussing chamber measurements of short-circuit currents (I_sc_) in freshly excised WT and CF rabbit tracheas. I_sc_ changes in response to amiloride (amil), forskolin/IBMX (Fsk/IBMX), GlyH101 (Gly101), and bumetanide (Bumet) are shown. Basal I_sc_ and transepithelial resistance were 49.82 ± 14.23 versus 57.74 ± 2.52 μA/cm^2^ and 60.07 ± 13.7 versus 60.66 ± 11.4 Ωcm^2^ for WT versus CF rabbits, respectively. *n* = 7 WT and 7 CF rabbits. *P < 0.05 versus WT rabbits. Unpaired, 2-tailed *t* test with Welch’s correction for unequal variance. (**F** and **G**) Ussing chamber measurements of I_sc_ for WT and CF rabbit tracheal epithelial cell cultures (**F**) (WT *n* = 3 and CF *n* = 2, 3–4 inserts/code), and normal and CF human bronchial epithelial cell cultures (**G**) (NL [*n* = 5] and CF [*n* = 1], 1717 1-G>A/N1303K compound mutation, no CFTR protein detectable, 3–4 inserts/code), illustrating responses to forskolin (Fsk), Vertex 770 (VX770), CFTR inhibitor 172 (I-172), and uridine triphosphate (UTP). *P < 0.05 versus WT cultures. Each dot represents the average of 3–4 inserts measurements. Unpaired, 2-tailed *t* test with Welch’s correction for unequal variance.

CFTR mRNA expression in the rabbit lower airway was robust and, importantly, extended from the trachea throughout the bronchi and bronchioles (Figure 6A and Supplemental Figure 8A). This distribution contrasts with the highly clustered and sparse distribution of CFTR mRNA expression in mouse large and small airways (Supplemental Figure 9). CFTR protein expression was examined in the tracheas of CF versus WT rabbits. Like human bronchial epithelia (HBE), robust CFTR bands B and C expression was observed in tracheal tissue extracts from WT rabbits by Western blot (Figure 6B and Supplemental Figure 8C). In contrast, CFTR protein was not detected in the tracheas of CF rabbits (line CFΔ1, Figure 6B and Supplemental Figure 8B; line CFΔ9, Supplemental Figure 8C). IHC confirmed CFTR localization in cells in which CFTR expression was abundant (e.g., in ionocytes in the nasal olfactory epithelia, the nasal SMG ducts, and in pancreatic collecting ducts), whereas no CFTR expression was detected in CF specimens (exemplified by CFΔ1 in Figure 6C). Like CF mice, ferrets, and pigs, but not sheep (10, 55–57), CF rabbit tracheas lacked the organized, concentric ring pattern characteristic of their age-matched WT counterparts (Figure 6D).

As a bioelectric characterization of CFTR lower airway function, Ussing chambers were employed to measure I_sc_ across CF and WT rabbit tracheas (exemplified by CFΔ1 in Figure 6E). A trend toward increased amiloride-sensitive I_sc_ was observed in CF compared with WT rabbits, whereas addition of Fsk and isobutylmethylxanthine (IBMX) to stimulate CFTR produced an increase in I_sc_ in WT but not CF rabbits. Similarly, apical addition of the CFTR inhibitor GlyH-101 decreased I_sc_ in WT but not CF rabbits. Finally, basolateral addition of bumetanide, an inhibitor of the Na^+^-K^+^-2Cl^–^ cotransporter, decreased I_sc_ in WT but not CF tracheas. These results are consistent with functional loss of CFTR-mediated anion secretion in the tracheas of CF rabbits, possibly associated with accelerated Na^+^ absorption.

We also tested whether rabbit CFTR was sensitive to pharmacologic CFTR modulators, as predicted by the high homology of rabbit and human CFTR. Chloride secretion was activated by human CFTR potentiator Vrtx 770 in primary airway cultures from WT rabbits, mimicking the response of non-CF human airway epithelial cell cultures (Figure 6, F and G; rabbit and human cultures, respectively). Notably, this response was absent in CF rabbit tracheas, suggesting that rabbits may be a useful model for CFTR pharmacotherapy (58).

**Figure 7:**
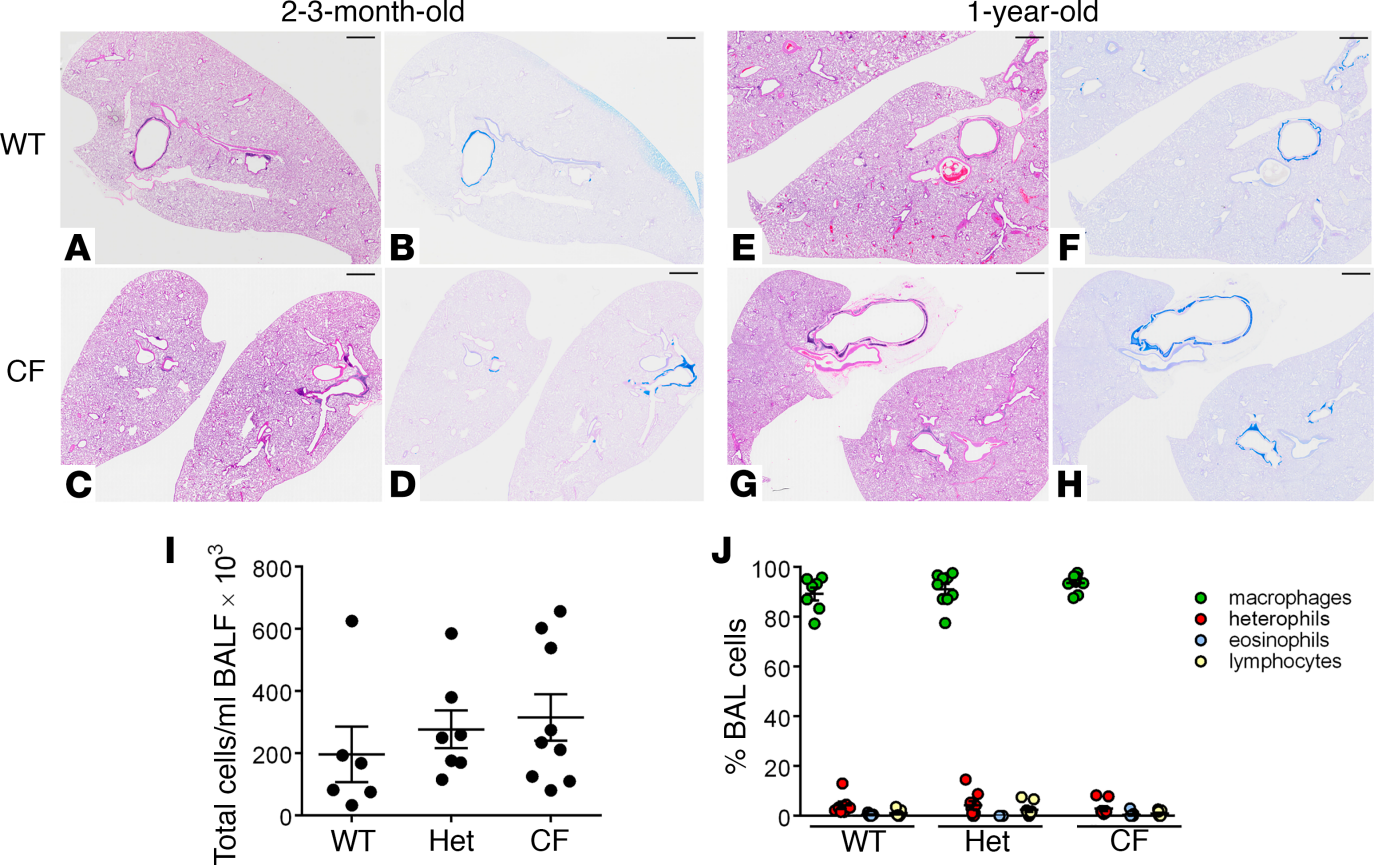
Characterization of lung phenotype in CF rabbits. (**A**–**H**) Representative micrographs of lung lobes from WT (**A**, **B**, **E**, and **F**) and CF rabbits (**C**, **D**, **G**, and **H**) harvested at approximately 2–3 months (**A**–**D**) or approximately 1 year (**E**–**H**) of age. No overt pathological changes were found in CF versus WT lungs at either age. Representative micrographs from *n* = 6–7 rabbits/genotype/age group. Scale bar: 2 mm. (**I**) Total cell recovered by bronchoalveolar lavage (BAL) in WT, Het, and CF rabbits (between 2 and 18 months of age), normalized by volume of fluid retrieved (BAL fluid). *n* = 6–9/genotype. (**J**) Differential cell counts in BAL from WT, Het, and CF rabbits (between 2 and 18 months of age). *n* = 6–9/genotype.

The incidence of spontaneous lung pathology was systematically evaluated by comparing CF rabbits with WT or Het littermates at UNC. Histopathological evaluation of 6 young (1- to 4-month-old) and 7 surviving (8- to 18-month-old) CF rabbits revealed no gross signs of muco-inflammatory lung disease (Figure 7, A–H). Consistent with these observations, bronchoalveolar lavage (BAL) total (Figure 7I) and differential (Figure 7J) cell counts were not different between CF and control rabbits. BAL bacterial cultures were routinely negative for both CF and control rabbits (*n* = 9 CF, *n* = 6 Het, and *n* = 6 WT). Molecular analysis of 16S ribosomal RNA sequences in a selected cohort of 1-year-old rabbits (*n* = 2 CF and *n* = 2 controls) revealed negligible bacterial loads (i.e., at or below the levels found in vehicle negative control) for all rabbits except for 1 control rabbit, which had a low-grade Pasteurella infection (also detected by standard culture method; data not shown).

**Figure 8:**
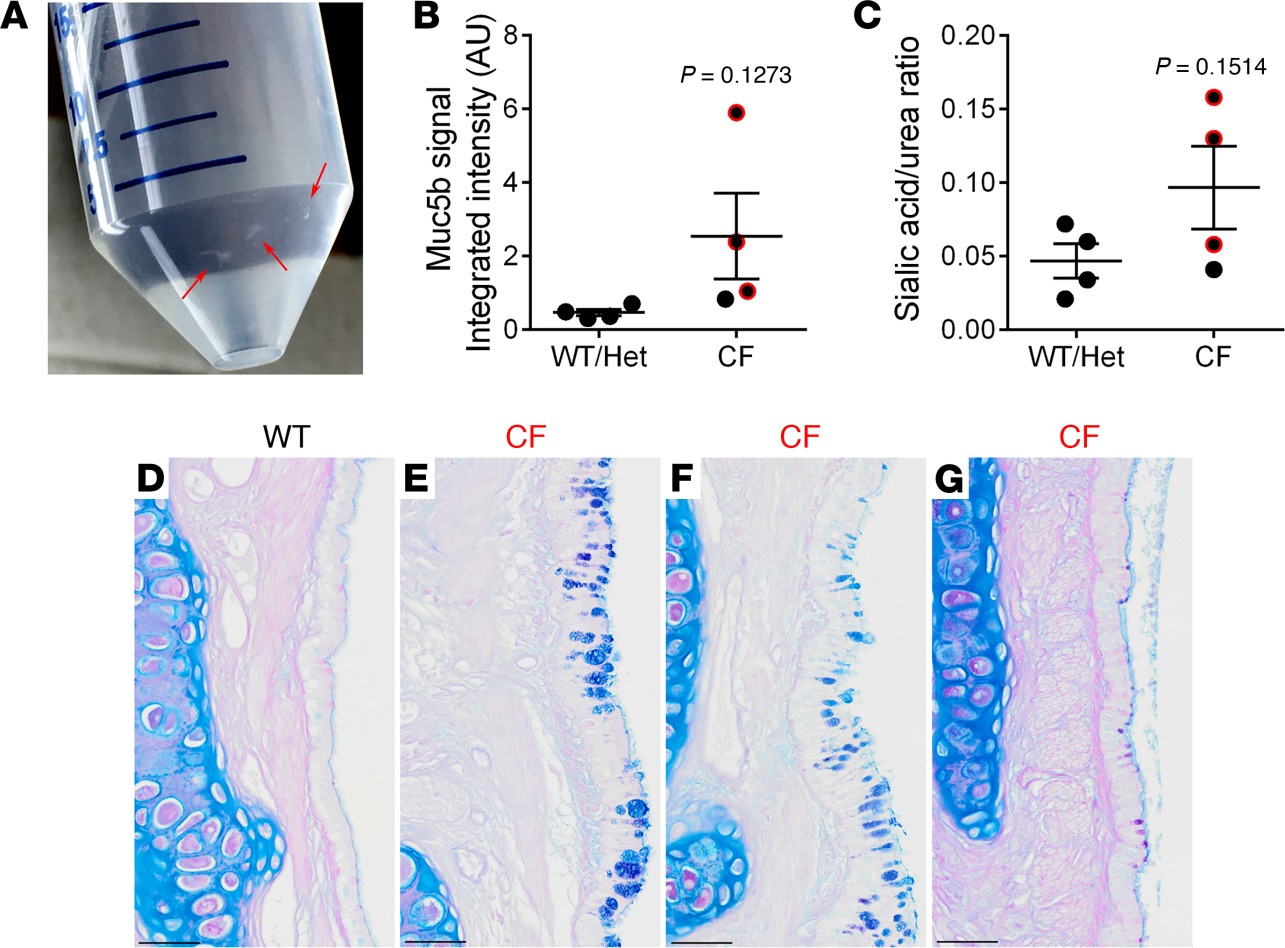
Subtle alterations in lung phenotypes in a fraction of surviving CF rabbits ≥ 1 year old. (**A**) Insoluble particles with mucus-like appearance were harvested in 3 of 5 surviving CF rabbits ≥ 1 year old. (**B** and **C**) Analysis of MUC5B agarose Western blot (**B**) and measurement of sialic acid/urea ratio (**C**) both indicated a trend toward higher mucin concentration in BAL isolated from these 3 surviving CF rabbits (symbol outlined in red) as compared with WT/Het littermates. (**D**–**G**) Histologically, the 3 surviving CF rabbits from which mucus flakes were isolated presented sparse, patchy areas of superficial epithelium mucus secretory cell metaplasia (**E**–**G**), as highlighted by AB-PAS stain versus WT rabbits (**D**). Scale bar: 50μm.

However, while collecting BAL from 1-year-old CF rabbits, visible “flakes” consistent with the appearance and consistency of small mucus plugs were observed (3 of 5 CF rabbits and 1 of 5 controls — i.e., the same control rabbit infected with Pasteurella mentioned above; Figure 8A). Although not significant due to the small sample size, a trend toward increased BAL Muc5b content, as measured by mucin agarose Western blot (Figure 8B) and sialic acid/urea ratio (Figure 8C), was also observed in BAL samples harvested from these 3 CF rabbits as compared with control WT/Het littermates. Moreover, histological evidence of spotty mucous cell metaplasia was found in the main stem bronchi of these 3 CF rabbits (Figure 8, D–G), suggesting that CF rabbit airways might exhibit early muco-inflammatory responses to external challenges, infectious or environmental, as compared with controls. As noted above, these abnormalities in CF rabbits were observed in the absence of bacterial infection, assessed by both culture and molecular methods.

Evidence for a potential role for an environmental challenge in producing CF lung disease arose from preliminary studies conducted on a cohort of Δ9 CF rabbit at WSU (*n* = 6 rabbits at P14–P35 and *n* = 11 rabbits at P44–P89). These studies utilized oral administration of Golytely through a syringe starting at P6 in an attempt to alleviate GI obstruction and reduce mortality. Five rabbits in this cohort (5 of 17, all at P44–P89) exhibited evidence of lung disease at sacrifice, including parenchymal consolidation (Supplemental Figure 10A) and the presence of intraluminal airway mucus at dissection (Supplemental Figure 10, B and C) and in BAL (Supplemental Figure 10D). Lung histologic studies revealed evidence of PAS-AB^+^ foreign material, with outlines consistent with plant cell walls, in the lungs of these 5 rabbits, that was accompanied by marked airway and parenchymal inflammation (Supplemental Figure 10E). These findings suggest that syringe feeding of Golytely caused sporadic aspiration of gastric/ingested content, and CF rabbits failed to clear aspirated material due to defective CFTR-dependent airway mucus clearance. Controlled studies comparing CF and WT rabbits will be required to test this notion in the future.

## Discussion

The rabbit was selected as a candidate CF model because it provides fidelity of CFTR expression in the lung with respect to humans, exhibits high CFTR homology to human CFTR, and is sensitive to potentiators developed for CF pharmacotherapy. Other important features of rabbits include the well-known breeding performance, early weaning (~2-months of age), a size intermediate between current models, and relatively affordable housing costs.

Our CRISPR/Cas9-mediated genetic modifications of rabbit CFTR have provided a useful model for studies of CF pathogenesis and therapy. One practical feature of the rabbit model is that female CF rabbits are fertile, making the generation of adequate study cohorts feasible. A second practical feature is that untreated CF rabbits do not exhibit perinatal MI and consequent early mortality observed in large CF animal models. Indeed, CF rabbits on a simple regimen of oral Golytely ad libitum have a median survival of 44 days, which could be extended to more than 80 days with physical examination–based pharmacologic interventions.

The mode of death in CF rabbits, although not the perinatal MI that affects several other CF animal models, did appear to reflect later-onset GI obstruction. Consistent with studies of freshly excised human tissues (59, 60), freshly excised rabbit jejunum exhibited the bioelectric properties predicted from loss of CFTR function (i.e., reduced Fsk-regulated anion secretion; Figure 1D). This defect is predicted to lead to reduced volume/water secretion in the gut, rendering CF intestinal secretions hyperconcentrated and hyperviscous. A second defect in CF jejunum was observed in young CF rabbits — namely, the increase in sodium-dependent glucose transport. This abnormality may reflect a host compensatory response to malnutrition and may have mixed effects on the CF rabbit GI tract (i.e., it may serve to facilitate glucose absorption but conversely may further exacerbate volume depletion and intestinal content hyperconcentration in the gut lumen).

Evidence of intraluminal accumulation of muco-fecal material was observed in 1- to 2-month-old CF rabbits, which could reflect problems in propulsion of hyperconcentrated, highly viscoelastic intraluminal material and/or gut dysmotility due in part to bacterial overgrowth. The elevated heterophil percentage in peripheral blood counts (Figure 2M) is consistent with persistent gut stasis and inflammation, which was strikingly exemplified by the progressive impaction of the vermiform appendix (Figure 2, A–J). Abnormal pancreatic acinar function, as suggested by low blood lipase levels (Figure 3I), may have also contributed to gut obstruction and the failure to thrive.

CF rabbits exhibit the classical nasal PD abnormalities associated with CFTR dysfunction at every age tested. Analysis of the upper airways in CF rabbits revealed abnormalities in both the olfactory and respiratory epithelium. With respect to the olfactory epithelium, a time-dependent degeneration/atrophy was observed in CF rabbits older than 9 months, similar to that reported in other models. Significant atrophy/mucus impaction of the Bowman glands underlying the olfactory epithelium and a marked submucosal lymphocytic inflammatory infiltrate were also observed (Figure 5 and Supplemental Figure 7). The olfactory pathology was associated with a CFTR expression pattern suggestive of ionocyte localization in this region.

CFTR expression in the rabbit nasal respiratory epithelium did not exhibit a focal, ionocyte-like pattern but had a more homogeneous distribution, resembling the lower respiratory tract. Nasal-associated lymphoid tissue–like (NALT-like) foci and diffuse infiltrates were identified underneath the respiratory epithelium, in areas where SMG were either not present and/or did not exhibit remodeling or impaction (Supplemental Figure 6). These findings may suggest abnormal intraluminal inflammatory stimuli, perhaps associated with adherent mucus (61).

There has been debate about the relative importance of SMG, which are found in the large but not small airway regions of the lung, in the initiation of CF lower airways disease. In human CF, the first detectable abnormalities in lung pathology, radiology, and pulmonary function appear to be in the small airways (62–65). However, these airways are more difficult to study in humans because of their protected/distal nature. The recent generation of a genetically modified pig model lacking SMG (EDA-KO pig; ref. 66) highlights how critical these studies are for our understanding of CF pathophysiology. In this context, the CF rabbit offers a suitable model to study the effect of CFTR functional depletion in airway compartments that are normally devoid of SMG (trachea and lungs) or containing SMG (nose and nasopharynx).

Unlike in mice (Supplemental Figure 9), CFTR expression in rabbit lower airways closely resembled that of humans and was detectable throughout distal airway regions (Figure 6A and Supplemental Figure 7). As noted above, CFTR expression in the lower respiratory epithelium was diffuse (i.e., not consistent with an ionocyte-clustered pattern). Expression of CFTR in the rabbit lower airway epithelia, which are highly enriched in nonciliated secretory cells (21) (i.e., club cells) is consistent with previous reports of a CFTR conductance in this cell type (67). Unfortunately, the tissue processing protocol required to perform RNAscope ISH precluded identification of specific cell types expressing CFTR (i.e., club cells) solely based on morphology. The data presented in Figure 6C suggest that identification of CFTR expressing cells by IHC would only detect high-expressing cells (e.g., ionocytes). However, colocalization with multiple cell marker RNAscope probes is a feasible future direction to identify the lower CFTR-expressing cells (i.e., club versus ciliated cells) in the rabbit lower airways.

Regardless of cell-specific localization, the bioelectric properties of the lower airways (i.e., trachea) in the CF rabbit mimicked the typical bioelectric properties of human CF subjects (i.e., complete absence of Fsk-mediated anion secretion). Importantly, rabbit tracheal CFTR was responsive to modulators active on human CFTR, suggesting that CF rabbits can be useful to study CFTR pharmacotherapy and, due to their size, are amenable to experimental in vivo gene therapy interventions.

With respect to lower airway pathogenesis, the absence of anion secretion, coupled with persistent or, possibly, raised sodium transport (i.e., increased amiloride-sensitive I_sc_) is predicted to dehydrate the airway surface, causing mucus hyperconcentration, mucus adhesion, and subsequent lung disease (68, 69). However, at least as assessed in this early observational study, CF rabbits failed to spontaneously develop overt lung disease (Figure 7). Longitudinal studies of human CF infants/children designed to capture the sequence of events downstream of CF specific ion transport defects have emerged from the AREST CF cohort (69, 70). These data have revealed that early CF lung disease is heterogeneous within the lung and with a relatively small fraction of CF neonates/young children exhibiting bacterial infection by 3 years of age. Importantly, a muco-inflammatory environment was the first detectable event in the human CF lung, followed by infection with anaerobes and subsequently the prototypic CF pathogens, including *P. aeruginosa* (69). The onset of human CF lung disease may be triggered by viral infections and/or gastric aspiration and coupled to vulnerable airway mucus clearance mechanisms in this period.

The lower airways of CF rabbits exhibited no spontaneous bacterial infection or heterophilic inflammation early in life. However, mucus “flakes” similar to those observed in the CF AREST human studies were observed in a fraction of 1-year-old CF rabbits. These CF rabbits were housed in a specific pathogen–free (SPF) facility that reduced/eliminated the possibility of intercurrent viral infections. A next step to test whether CF rabbits exhibit vulnerability to inhaled/aspirated foreign insults, similar to early human CF lung disease, is to raise rabbits in wild-like environments and/or expose them to experimental challenges such as intermittent lower airway viral infections or controlled aspiration.

In summary, the CF rabbit model offers the CF research community an intermediate-sized model that is relevant to human CF pathogenesis and therapy. In particular, CF rabbits exhibit robust readouts of CFTR function for immediate testing of pharmacotherapy/genetic therapy, including: (a) the classic NPD measurement and spontaneous development of upper airway pathology; (b) abnormal GI bioelectric properties coupled to weight loss and mortality; (c) a CFTR protein responsive to human CFTR modulators, including VX770; and (d) lower airway bronchiolar CFTR expression and abnormal bioelectric properties. Future improvements in animal husbandry may make the rabbit model even more useful, including gut-corrected rabbits, lung-specific CFTR KO, or F508del rabbits that may be maintained on modulators to make them available for CF-relevant challenges. Moreover, the currently available CF rabbit lines could be used to investigate the preterm-to-term development of CF intestinal disease, congenital bilateral absence of vas deference (CBAVD), pancreatic disease, and upper airway disease, all without the complication of MI (Supplemental Table 4).

## Methods

### CRISPR/Cas9 construction and RNA synthesis.

A robust CRISPR/Cas9 gene-editing platform developed in NZW rabbits was used (22, 24, 71), utilizing the Cas9 expression plasmid JDS246 and sgRNA expression plasmid DR274 (Addgene). The sgRNA (GGAGAGTTGGAGCCTTCAGA), located on exon 11 of the rabbit CFTR gene, 87 bp upstream of the F508 locus, was designed using Zifit software (http://zifit.partners.org/ZiFiT/; Supplemental Figure 1A). Microinjection, embryo transfer, and confirmation of gene targeting events were performed as previously described (24).

### Generation of CFTR-KO rabbits.

A total of 162 embryos were transferred to 6 recipients, 11 live kits were obtained, and 3 kits were confirmed as CFTR-KO founders (Supplemental Figure 1B). Upon sexual maturation, CFTR-KO founder (F0) rabbits were bred with WT counterparts to generate F1 generation animals. Newborn kits were genotyped, and the ones carrying indels in 1 allele were identified as F1 Het rabbits. F1 Het rabbits were then interbred to produce homozygous CFTR-KO rabbits in the F2 and later generations. To determine genotypes, ear skin tissues were biopsied, and genomic DNA was extracted. Genomic DNAs were used for PCR with the primer set F1/R1 (F1: 5′-GCTTTATGGTTCCCTTACGGTTTA-3′, R1: 5′-GACTTAACCTGCTTCACCACAA-3′). PCR products were purified and sequenced for detection of indel mutations proximate to the sgRNA target sequence. On the chromatographic curves, peaks on peaks approximating the targeting site indicated an indel event. Mutation Surveyor (Softgenetics) was used to analyze sequencing results.

To test whether CFTR mRNA was still expressed in the mutant CFΔ1 line, quantitative PCR (qPCR) and mRNA ISH were conducted on a small number of animals, and both revealed detectable CFTR mRNA in both CF and WT rabbits (Supplemental Figure 11A for qPCR in WT and CF trachea and Supplemental Figure 11B for mRNA ISH in CF jejunum).

### Off-target analyses.

Using the sgRNAs for the KO experimental and all 4 possible PAM sequences NGG (AGG, TGG, CGG, and GGG) at the 3′ end, BLAST analysis of the rabbit genome was performed on the NCBI website (https://blast.ncbi.nlm.nih.gov/Blast.cgi?PAGE_TYPE=BlastSearch&PROG_DEF=blastn&BLAST_PROG_DEF=megaBlast&BLAST_SPEC=OGP__9986__12818). A total of 51 potential off-targets were identified, and the top 20 sites were selected for off-target analysis. Twenty sets of primers (Supplemental Table 1) were designed to amplify these potential off-target sites from the genomic DNA isolated from 3 CF rabbits. The PCR products were sequenced (using the sequence primers showed in Supplemental Table 1) to detect off-target events, and no mutations were detected (Supplemental Table 2).

### Animal husbandry.

Rabbit colonies were maintained in dedicated Association for Assessment and Accreditation of Laboratory Animal Care International–certified (AALAC-certified) facilities at WSU (lines CFΔ1, CF+1, and CFΔ9), UM (lines CFΔ1, CFΔ9, and CF+1), and UNC (line CFΔ1). Rabbits were housed in SPF facilities, on a 12-hour day/night cycle. At UNC, at 2 weeks of age, the dam and kits were given Golytely (an osmotic laxative, Braintree Labs) to drink instead of water. The CF rabbits consumed this laxative for their entire life. At 4 weeks of age, all kits were given Rabbit Liquid diet Bio-Serve (Bio-Serve) in a bowl twice a day as a supplement to their regular rabbit chow (Teklad Global rabbit diet 2030, Envigo). Rabbit diet was also integrated with vegetables (cabbage, carrots, celery, romaine lettuce) 3 times a week and fruit (apples, grapes) once a week. At UM, the main diet was Laboratory Rabbit Diet #5321 (LabDiet). At 1 week of age, all CF rabbits are weighed and palpated every other day for abdominal masses. If masses were detected, rabbits were treated twice a day with: 50 mL subcute warmed physiological saline; oral 0.5 mg/kg cisapride (increases motility of lower gut; Roadrunner Pharmacy); oral 0.5 mL/kg simethicone (anti-gas agent; MedTech Products); oral 0.5 mg/kg metoclopramide (increases motility of small bowel; Roadrunner Pharmacy); and oral 0.5 mL/kg lactulose (a laxative; Actavis). Treatment continued until masses resolved. Some of the older CF rabbits presented multiple times with gut symptoms, which were reversed with our treatment protocol. Using this strategy, we were able to raise 6 CF rabbits to ≥ 1 year of age. Cages were supplemented with fresh hay (101.105.5000, Oxbow Animal Health), and rabbits were given active, differentiated playtime in a large enclosure, twice a week.

### Immunoblotting.

For CFTR Western blot, tracheal tissues from CF and WT rabbits were homogenized in lysis buffer (50 mM HEPES [pH 7.4; MilliporeSigma], 150 mM NaCl [MilliporeSigma], 1 mM EDTA [MilliporeSigma], 1% NP-40 [MilliporeSigma], 10% glycerol [MilliporeSigma] with protease inhibitors cocktail Roche; catalog 11836145001) and centrifuged for 10 minutes at 10,621*g*, 4°C. Total protein concentration was assessed by protein assay (Bio-Rad, 500-0006), and 50 μg total protein was resuspended in 6× SDS sample buffer and denatured at 42°C for 10 minutes. Samples were resolved by SDS-PAGE, transferred to PVDF [Thermo Fisher Scientific] membranes, and blocked at room temperature for 1 hour with 5% (w/v) skim milk in TBST (10 mM Tris [pH 8.0; MilliporeSigma], 150 mM NaCl, 0.05% Tween 20 [MilliporeSigma]). Membranes were incubated with primary antibodies (Ab 596 from J. Riordan, UNC; ref. 72) 1:5000 in TBST with 10% FBS at room temperature for 1 hour, washed 4 times with TBST, and incubated with horseradish peroxidase–conjugated secondary antibodies (1:5000, ab205719, Abcam) in TBST with 10% FBS for 1 hour, followed by 5 washes with TBST. The reactive bands were visualized using enhanced chemiluminescence substrate (PerkinElmer). CFBE41o- WT CFTR cell lysate was used as the positive control. For MUC5B detection in BAL samples, agarose Western blot was performed as previously described (61), using a custom-made polyclonal goat antibody generated against an immunogenic peptide of mouse Muc5b previously used to successfully generate the UNC 222 rabbit polyclonal antibody against murine Muc5b (73). Validation of this reagent was carried out using BAL samples, mucus-enriched samples isolated from the nasopharyngeal cavity of rabbits, and by comparison with the migration pattern of murine Muc5b in agarose gels, as illustrated in Supplemental Figure 11C. See complete unedited blots in the supplemental material.

### Immunohistochemical localization of CFTR.

Tissues (airway and olfactory epithelia isolated ex vivo from the nasal cavity, trachea, lungs, and duodenum/distal colon/pancreas isolated en bloc) were immersion fixed in 10% neutral-buffered formalin (NBF, Thermo Fisher Scientific) for 48–72 hours. Paraffin-embedded sections were incubated at 65°C for 16 hours, and deparaffinized with xylene (3 changes × 10 minutes; Richard-Allan Scientific) and graded ethanol (100%, 2 × 5 minutes; 95%, 1 × 5 minutes; 70%, 1 × 5 minutes; Thermo Fisher Scientific). After rehydration in phosphate buffered saline (PBS; pH 7.2), endogenous peroxidase was quenched with 0.5% H_2_O_2_ (Thermo Fisher Scientific) in methanol for 15 minutes at room temperature under gentle rocking. Slides were washed in PBS, and antigen retrieval was performed by boiling the slides in 0.1M sodium citrate, pH 6.00 (3 cycles with microwave settings of 100% power for 6.5 minutes, 60% for 6 minutes, and 60% for 6 minutes, refilling the Coplin jars with deionized water after each cycle). After cooling for 15 minutes and rinsing with dH_2_O (1×) and PBS (1×), slides were blocked with ready-to-use Blocking One Histo (Nacalai USA Inc., 06349-64) for 2 hours at room temperature. Primary antibody (mouse anti–human Ab 528 from J. Riordan, UNC; ref. 72) was diluted 1:1000 in Blocking One Histo and incubated over night at 4°C. Sections were washed in PBS and secondary antibody (biotinylated goat anti-mouse IgG, Jackson ImmunoResearch, at 1:200 dilution in 5% Blocking One Histo/PBS) was applied for 1 hour at room temperature. After washing in PBST, slides were incubated with avidin-peroxidase complex according to the manufacturer instruction (Vectastain kit, Vector Laboratories) and washed in PBS. Detection was performed using metal-enhanced DAB as chromogenic substrate. DAB solution was prepared by dissolving 0.095 g DAB in 200 mL of 0.1M sodium acetate (MilliporeSigma), 140 mM NaCl, and 65 mM NiSO_4_ (H_2_O)_6_ and filtered through #1 Whatman paper. Right before use, fresh 30% H_2_O_2_ was added to DAB solution to 0.004% final concentration (i.e., 25 μL 30% H_2_O_2_ in 200 mL DAB solution). Slides were sequentially incubated in (a) 0.1M sodium acetate for 1 minutes; (b) DAB solution plus H_2_O_2_ for 4 minutes; (c) 154 mM NaCl in 0.1M Tris HCl (pH 7.6) for 1 minute; and (d) 21 mM cobalt chloride (CoCl_2_[H_2_0]_6_; MilliporeSigma) in 0.05M Tris HCl (pH 7.2) for 4 minutes. Slides were quickly rinsed in deionized water and counterstained with nuclear Fast Red (0.1% nuclear fast red in 5% aluminum sulfate; MilliporeSigma) for 4 minutes, rinsed under deionized water until cleared, and dehydrated through ethanol (70%, 95%, and 100%) and xylene for 2 minutes each. Coverslipped slides were imaged by transmitted light microscopy, using an Olympus BX60 microscope with an UPlanFI 100×/1.3 NA lens.

### Measurements of I_sc_ by Ussing chamber.

I_sc_ recordings in Ussing chambers were conducted on ex vivo intestinal and tracheal preparations, as well as in vitro airway epithelial cell cultures, according to previously published protocols (36, 46, 53). Specifically, the intestinal preparations were studied as previously described (36), with the exception that the serosal muscle and connective tissue were stripped from the preparation. After equilibration, Fsk (1 × 10^–5^ M, bilateral), bumetanide (1 × 10^–4^ M, basolateral), glucose (5 mM, apical), and phloridzin (1 × 10^–4^ M, apical; MilliporeSigma) were sequentially added and changes in I_sc_ measured. Tracheal preparations were prepared as 4 cm–long sections excised and immediately placed into Ringer’s buffer (120 mM NaCl, 25 mM NaHCO_3_, 3.3 mM KH_2_PO_4_, 0.8 mM K_2_HPO_4_, 1.2 mM MgCl_2_, 1.2 mM CaCl_2_, and 10 mM glucose) with 10 μM indomethacin (MilliporeSigma), that was continuously gassed with 95% O_2_ and 5% CO_2_ for 10 minutes. After incubation, connective tissues were removed from the trachea under a dissection microscope. A longitudinal cut was made along the trachea, exposing the mucosal side. The tracheal epithelium was carefully placed on a slider with an exposed surface area of 0.20 cm^2^. Both sides of the epithelium were perfused with equal amounts of Ringer’s buffer at 37°C and gassed with 95% O_2_ and 5% CO_2_, providing gas lift circulation. Each sample was equilibrated under voltage clamp (I_sc_) conditions for 20 minutes. Once a baseline I_sc_ was achieved, drugs were added as follows: amiloride (apical, 1 × 10^–5^ M), Fsk (basolateral, 1 × 10^–5^ M), GlyH101 (apical, 1 × 10^–3^ M, provided by R. Bridges, Chicago Medical School, North Chicago, Illinois, USA), and bumetanide (basolateral, 1 × 10^–3^ M). Isolation of airway epithelial cells from rabbit trachea to generate primary cultures was performed according to published protocol (74), with the following modifications: the connective tissue surrounding freshly excised tracheas was removed by fine dissection, and the trachea was cut in short segments and opened longitudinally to expose the airway epithelium. Tracheal segments were placed in digestion solution (1% Protease XIV with DNase; amphotericin [MilliporeSigma], 1:200; gentamicin [MilliporeSigma], 1:1000; in F12 medium [Thermo Fisher Scientific]) on a rocker in cold room overnight (4°C). Declumping was performed for 15 minutes using 1–3 mL of Accutase Cell Detachment Solution (Innovative Cell Technologies) at 37°C, and cells were seeded on 12 mm Millicell Cell Culture Inserts (MilliporeSigma) and grown for about 21 days until air-liquid interface was established. Ussing chamber electrophysiology was performed on these cultures according to published protocols (75–77).

### In vivo NPD measurement.

Rabbit nasal PD measurements were conducted identically to those published for the mouse (53), except that the rabbit was anesthetized by inhalation of 3% isoflurane (Piramal Healthcare) and the perfusate flow rate was 50 μL/min.

### Histology.

Tissues were fixed in 10% NBF for at least 24 hours, embedded into paraffin blocks, and cut into 5 μm sections. Heads were dissected, and lower jaw, brain, and ocular bulbs were removed to allow uniform fixation and decalcification of the skull. Rabbit heads were fixed and decalcified using Formical-4 (StatLab Medical Products) for several weeks, whereas mouse heads were fixed in 10% NBF for 48 hours and decalcified with Immunocal (Decal Chemical Corp.) for 24 hours. Following decalcification, the nasal cavity was cut at selected level as previously described (78, 79). Fixed specimens were embedded, sectioned, and stained with H&E, AB-PAS, or Masson’s trichrome stain. Tissue blocks received a numerical code at time of embedding, and scoring of the slides was performed by an investigator blinded to specimen genotype.

### BAL collection.

Animals were anesthetized with 3%–5% isoflurane inhalation and euthanized by exsanguination and thoracotomy. The chest cavity was open, and the left lobes were ligated at the level of the extrapulmonary bronchus to preserve intraluminal content. BAL was performed on the right lobes by instilling and retrieving sterile PBS through a custom-made sterile cannula held in place by a suture. The right lobes were lavaged 3 consecutive times, with a first volume of 10 mL PBS (retrieved volume approximately 7 mL) followed by a second volume of 20 mL PBS, also repeated 3 times (retrieved volume, about 18 mL). Fractions from the first and second lavage were pooled and processed for total and differential cell counts, microbiological analyses, mucin Western blot, or sialic acid/urea assay, as previously described (61, 80, 81). Due to the BAL dilution, samples for microbiology were concentrated by centrifugation (i.e., 1 mL aliquots of unfractionated BAL were spun at maximum speed in a tabletop centrifuge (21,000*g*) at 4°C for 10 minutes), the supernatant was aspirated to 100 μL, and the pellet was resuspended before serially plating on Columbia anaerobic blood agar (BD Biosciences, 221928) or processing for DNA isolation and 16S rRNA gene quantification by PCR and sequence analysis (80).

### mRNA ISH.

To obtain tissues for mRNA ISH using the RNAscope technology (Advanced Cell Diagnostics), animals were anesthetized with 3%–5% isoflurane inhalation and euthanized by exsanguination and thoracotomy. Lungs were inflation fixed with 10% NBF, and 1–2 cm × 0.5 cm excised samples of other organs were immersion fixed in 10% NBF (25–50 times the volume of tissue) for 48 hours at room temperature, under gentle oscillation. Tissues were rinsed by 3 consecutive 30-minute incubations in distilled water and transferred to 70% ethanol until further processing for paraffin embedding and sectioning. RNA ISH for CFTR was performed on 5 μm tissue sections with RNAscope 2.5 HD Reagent Kit according to the manufacturer’s instructions as previously published (82), with the main modification being an initial overnight incubation of the slides at 65°C to promote adhesion of the paraffin sections to the glass slide. CFTR was detected using custom probes for rabbit (Oc-CFTR, 497241) and mouse (Mm-Cftr, 483011) sequences (ACDBio). The rabbit housekeeping gene peptidylprolyl isomerase B (Oc-PPIB, 406751) was used as positive control to test for RNA quality in rabbit tissues, and mouse Ubiquitin C (Mm-Ubc, 310771) was used for mouse tissues. The bacterial gene 4-hydroxy-tetrahydrodipicolinate reductase (DapB, 310043) was used as a negative control in both rabbit and mouse tissues. CFTR mRNA ISH was performed at UNC on tissues from approximately 3-month-old WT NZW rabbits (The Charles River Laboratory) and 6-week-old C57BL/6N mice (Taconic).

### Metabolic panel and hematological analyses.

Animal clinical laboratory analyses were performed by the Animal Histopathology & Laboratory Medicine Core at the University of North Carolina. Complete blood counts (CBCs) were performed using a veterinary-specific hematology analyzer (IDEXX ProCyte DX Hematology Analyzer, IDEXX Laboratories) using rabbit species specifications for recognizing the size and density of each cell type. Serum testing was conducted on an Alfa Wasserman Vet Axcel clinical chemistry analyzer using manufacturer-specific reagents for respective tests.

### Statistics.

Survival curves were compared using Logrank test (Mantel-Cox, with and without the Gehan-Breslow-Wilcoxon test for extra weight on early time points) using GraphPad Prism 8 (GraphPad Software) with *P* < 0.05. Body weights were compared by 2-way ANOVA followed by Tukey’s post hoc test for multiple comparisons, with *P* < 0.05. For bioelectric studies, an unpaired, 2-tailed *t* test was used to determine if there was a significant difference between genotypes, with *P* < 0.05, and Welch’s correction was applied whenever *F* test revealed unequal variance groups. Mean ± SEM is shown in all figures, along with individual data (each dot represents 1 animal or cell culture donor tissue).

### Study approval.

All animal maintenance, care, and use procedures were reviewed and approved by the University Committee on the Use and Care of Animals of the UM, the IACUC of WSU, and the IACUC at the UNC. All experiments were performed according to the principles outlined by the Animal Welfare and the NIH *Guide for the Care and Use of Laboratory Animals* (National Academies Press, 2011).

## Author contributions

JX, JZ, JS, JR, DY, and XL generated CFTR-KO rabbits, managed the colony, performed sample collection, and conducted characterization experiments at UM. XH, CR, HJ, HGW, MB, QW, XC, MS, HW, YQ, YX, K. Zhang, RB, SM, LH, and FS managed the rabbit colony, performed sample collection, and conducted characterization experiments at WSU. HM conducted characterization experiments in the airways. K. Zaman conducted molecular characterization experiments. RAF conducted characterization experiments in the airways and GI tissues. BRG and TDR managed the UNC rabbit colony, designed the experiments and treatment plans, and performed health assessment, sample collection, histological analyses, electrophysiological measurements, and data analysis. ALB and KJW performed BAL studies and collection of samples for histology. RCG, ALB, and WKO performed and analyzed RNAscope ISH assays. CRE performed sialic acid/urea assay; MG supervised performance of in vitro electrophysiological assays. ALB performed mucin agarose Western blots, histopathology, and data analysis. YEC and FS conceived the idea. ALB, JX, XH, CR, and XL prepared the figures. ALB, JX, RCB, XH, CR, YEC, and FS wrote the manuscript.

## Supplementary Material

Supplemental data

## Version 1 11/24/2020

In-Press Preview

## Version 2 01/11/2021

Electronic publication
