# Supplementary material for: Phenotypes of CF rabbits generated by CRISPR/Cas9-mediated disruption of the CFTR gene
Source: JCI Insight. 2021 Jan 11;6(1):e139813. doi: 10.1172/jci.insight.139813 (PMC7821608; doi:10.1172/jci.insight.139813)
Supplement: Supplemental data [file jciinsight-6-139813-s063.pdf]

Title: Phenotypes of CF rabbits generated by CRISPR/Cas9-mediated disruption of the CFTR gene

Authors:

Jie Xu[a,1], Alessandra Livraghi-Butrico[b,1], Xia Hou[c,1], Carthic Rajagopalan[c,1], Jifeng Zhang[a], Jun Song[a], Hong Jiang[c], Hong-Guang Wei[c], Hui Wang[d], Mohamad Bouhamdan[c], Jinxue Ruan[a], Dongshan Yang[a], Yining Qiu[e], Xie Youming[d], Ronald Barrett[f], Sharon McClellan[f], Hongmei Mou[g], Qingtian Wu[c], Xuequn Chen[c], Troy D. Rogers[b], Kristen J. Wilkinson[b], Rodney C. Gilmore[b], Charles R. Esther Jr.[b], Khalequz Zaman[h], Xiubin Liang[a], Michael Sobolic[c], Linda Hazlett[f], Kezhong Zhang[e], Raymond A. Frizzell[i], Martina Gentzsch[b], Wanda K. O'Neal[b], Barbara R. Grubb[b], Y. Eugene Chen[a], Richard C. Boucher[b], Fei Sun[c#]

SUPPLEMENTAL MATERIAL

Supplemental Figure 1. **Production of CFTR KO rabbits**. **A**) Illustration of sgRNA-02 and CRISPR/Cas9. **B**) Embryo transfer results for production of CFTR mutant rabbits. **C**) Genotypes of CFTR mutant lines. Specific insertions and deletions (indels) are shown. **D**) Breeding results of F1 generation CFΔ1 rabbits.

Supplemental Figure 2. **CF rabbit male reproductive tract abnormalities**. Male reproductive tract of wild-type (WT) and CF rabbits (~ 300 days-old). Notable is the complete absence of epididymis and vas deferens in CF rabbit.

Supplemental Figure 3. Additional characterization of CF rabbit GI phenotypes. A) Overall appearance of WT (+/+) and a CFΔ1 (-/-) rabbit at 30 days of age, illustrating the smaller size of

CF rabbits as compared to WT littermates. **B**) Complete CFTR RNAscope® panel (including CFTR, Negative, and Positive mRNA probes, red chromogen) for WT rabbit jejunum and distal colon. Scale bar 50µm. **C**) Ussing chamber characterization of freshly excised jejunal tissue from WT (black) and CF (gray) rabbits (mean PND 367), illustrating short circuit currents (Isc) under basal conditions, forskolin (10^{-5} M) stimulation, bumetanide (10^{-4} M) inhibition, glucose (5mM, apical) addition, and phloridzin (10^{-4} M) inhibition. Note the absence of increased phloridzin response in older CF rabbit in stable health conditions, as compared to the one shown in Figure **1D** for younger rabbits. N = 3-4/genotype *$p < 0.05$ different than WT. Unpaired, two-tailed t-test with Welch's correction for unequal variance.

Supplemental Figure 4. Additional characterization of WT and CF rabbit blood metabolic panel and liver histopathology. A-K) Blood Chemistry panel for liver, pancreatic, and renal parameters in ~1 year-old WT and Δ1 CF rabbits raised at UNC (n=4-7/genotype). Abbreviations: Alanine aminotransferase (ALT), alkaline phosphatase (ACP), aspartate aminotransferase (AST), gamma-glutamyl transpeptidase (GGT), lactic acid dehydrogenase (LDH), blood urea nitrogen (BUN). * $p < 0.05$ CF vs. WT, unpaired, two-tailed t-test. **L**) Representative histological micrographs of WT and CF rabbit liver, stained for collagen (Masson's trichrome) or H&E, centered on main portal triads. Scale bar = 0.1mm. Representative micrographs from n=3 CF and control rabbits.

Supplemental Figure 5. **CFTR mRNA expression in WT rabbit pancreas and pancreas histopathology. A**) Complete H&E and CFTR RNAscope® panel (including CFTR, Negative,

and Positive mRNA probes) for WT rabbit (red chromogen) and WT mouse (teal chromogen) pancreas. Scale bar = 20μm. Representative micrographs from n=2 rabbits and 2 mice. **B**) Representative histological micrographs of the focal lesion observed in the pancreas of 3 out of 5 Δ9 CF rabbits at UM. Region highlighted by boxes in the left panels (scale bar 0.2mm) are presented at higher magnification in the right panels (scale bar 0.1mm). Asterisks indicate areas of exocrine gland distension and fibrosis. Arrows indicate inflammatory infiltration. H&E stain. **C**) Representative histological micrographs of CF and WT rabbit pancreas from the UNC Δ1 cohort, where no pathological changes were observed. Scale bar = 0.1mm. H&E stain. Representative micrographs from n=6-7 CF and control rabbits.

Supplemental Figure 6. **Characterization of CFTR expression in the murine nasal cavity.** **A**) Complete H&E, AB-PAS, and CFTR RNAscope® panel (including CFTR, Negative, and Positive mRNA probes, red chromogen) for respiratory and olfactory epithelia in the nasal cavity of WT mice. Scale bar = 0.1 mm. **B-C**) High power magnification of mouse respiratory (**B**) and olfactory (**C**) nasal epithelium region of interest highlighted with boxes in the low-magnification images above. Note the clustered distribution of CFTR in both of these regions. Scale bar= 20μm. Representative micrographs from n=2 mice.

Supplemental Figure 7. **Nose pathology in CF rabbits.** Additional evidence of inflammatory remodeling in the olfactory **(A-D)** and respiratory **(E-H)** mucosa of CF **(B, D, F, H)** vs. WT **(A, C, E, G)** rabbit nose. Scale bar = 0.1mm

Supplemental Figure 8. **CFTR mRNA expression in WT rabbit lower airways. A**) Complete H&E, AB-PAS, and CFTR RNAscope® panel (including CFTR, Negative, and Positive mRNA probes, red chromogen) for the lower airways of WT rabbits (~ 3 months-old). Scale bar 20μm. Representative micrographs from n=2 rabbits. **B-C**) Uncropped western blots for CFTR and βactin on HBE and tracheal tissue lysates from WT and CF rabbit line Δ1 (**B,** shown in Figure 6B) and line Δ9 (**C**) with labels for protein markers' molecular weight.

Supplemental Figure 9. **CFTR mRNA expression in WT mouse lower airways.** Complete CFTR RNAscope® panel (including CFTR, Negative, and Positive mRNA probes, red chromogen) for the trachea, large and small airways of WT mice (PND56). Arrows indicate CFTR-positive cells. Scale bar 20μm. Representative micrographs from n=2 mice.

Supplemental Figure 10. Evidence of unresolved aspiration in a subset of CF rabbits after oral Golytely administration starting at PND6.

A) Gross appearance of lungs from a subset of CF rabbit syringe-fed with Golytely starting at PND6. Note the consolidated/yellowish appearance of the lung parenchyma (arrow). **B-D**) Strings of mucus-like material could be pulled out of the airways (**B**, arrow), occasionally contained particulate yellow/green material (**C**, dotted line highlights the main stem bronchus circumference), and could be isolated by BAL (**D**, arrows. The presence of blood in BAL suggests lung injury). **E**) Representative micrographs of lung sections stained with H&E, Periodic-Acid Shiff and Alcian blue, illustrating the presence of particles deposited in the airway and alveolar spaces (arrows), along with airway (*) and parenchymal (#) inflammatory cell

infiltration in the lungs of a subset of CF rabbits undergoing oral Golytely administration starting at PND6.

Supplemental Figure 11. CFTR mRNA expression in CF rabbit tissues and rabbit MUC5B agarose western blot. A) CFTR mRNA expression in the lungs of WT vs. CF rabbits (line Δ1) as assessed by qPCR. Unpaired, two-tailed t-test p=0.12. **B**) Complete CFTR RNAscope® panel (including CFTR, Negative, and Positive mRNA probes, red chromogen) for CF rabbit jejunum, indicating that CFTR mRNA is still present and detectable in specimens harvested from CF Δ1 rabbits. Scale bar = 0.1mm. Pancreas, proximal colon, trachea, and lungs from the same CF rabbit were also probed, yielding similar results. **C**) Validation of a goat polyclonal antibody to probe for MUC5B in rabbit bronchoalveolar lavage samples. WT (lanes 1-4) and CF (lanes 4-8) samples (unfractionated/whole BAL, BAL supernatant and pellet fractions obtained after low speed centrifugation) were run under reduced conditions and probed with a polyclonal goat antibody raised against an immunogenic peptide of mouse Muc5b (See Methods). Lane 9-12 contain samples not discussed in the current manuscript. Asterisks indicate samples of concentrated mucus harvested from the nasopharyngeal region of WT (black and blue asterisks) or CF (red and yellow asterisks) rabbits, which was used as a positive control, given the extreme dilution of the BAL samples. The lanes marked with green asterisks contain BAL sample harvested from WT mice. This control was used to verify high molecular weight migration of the putative rabbit MUC5B band, and to distinguish it from aspecific low-molecular bands. Rabbit MUC5B appears to migrate at a slightly higher MW compared to murine MUC5B.

A

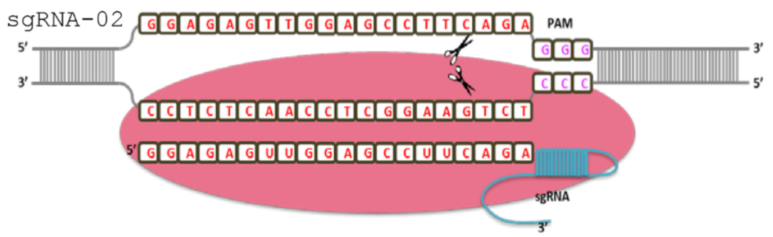

B

#embryos transferred	#recipients	#kits born	#positive F0 KO (%)
162	6	11	3 (27)

C

Animal (sex)	Sequence	Genotype
Control	GATGATGATTATGGGAGAGTTGGAGCCTTCAGA**GGG**TAAAATTAAGCACAGTGGAAG	WT
F0#1(F)	GATGATGATTA---------------------------------AGCACAGTGGAAG<br>GATGATGATTATGGGAGAGTTGGAGCCTTCTAGA**GGG**TAAAATTAAGCACAGTGGAAG	Δ33<br>+1
F0#2(M)	GATGATGATTATGGGAGAGTTGGAGCCTT-AGA**GGG**TAAAATTAAGCACAGTGGAAG	Δ1
F0#3(F)	GATGATGATTATGGGAGAGTTGGAGCCTT--**CCAACTCTGGAAGA**-CACAGTGGAAG<br>GATGATGATTATGGGAGAGTTG---------GA**GGG**TAAAATTAAGCACAGTGGAAG	Δ17+14<br>Δ9

D

Litters bred	Total born	# Genotypes (%)		
		WT	CFTR+/-	CFTR-/-
28	186	48 (25.8)	96 (51.6)	42 (22.6)

Supplemental Figure 1. **Production of CFTR KO rabbits**. **A**) Illustration of sgRNA-02 and CRISPR/Cas9. **B**) Embryo transfer results for production of CFTR mutant rabbits. **C**) Genotypes of CFTR mutant lines. Specific insertions and deletions (indels) are shown. **D**) Breeding results of F1 generation CFΔ1 rabbits.

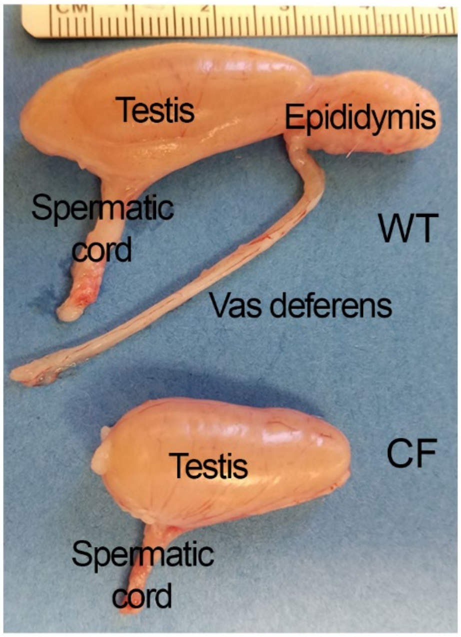

Supplemental Figure 2. **CF rabbit male reproductive tract abnormalities.** Male reproductive tract of wild-type (WT) and CF rabbits (~ 300 days-old). Notable is the complete absence of epididymis and vas deferens in CF rabbit.

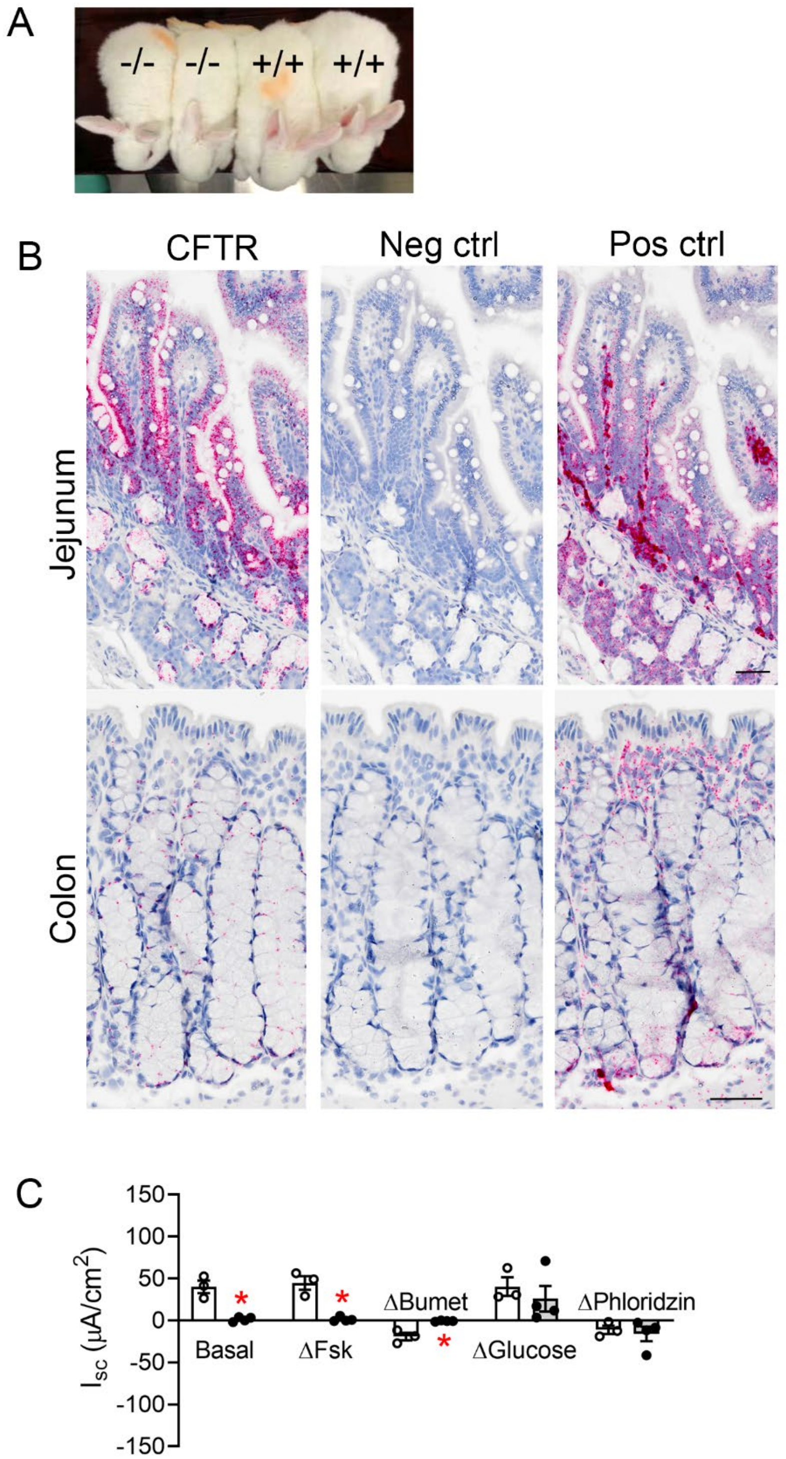

Supplemental Figure 3. Additional characterization of CF rabbit GI phenotypes. A) Overall appearance of WT (+/+) and a CFΔ1 (-/-) rabbit at 30 days of age, illustrating the smaller size of CF rabbits as compared to WT littermates. **B**) Complete CFTR RNAscope® panel (including CFTR, Negative, and Positive mRNA probes, red chromogen, See Methods for details) for WT rabbit jejunum and distal colon. Scale bar 50µm. **C**) Ussing chamber characterization of freshly excised jejunal tissue from WT (black) and CF (gray) rabbits (mean PND 367), illustrating short circuit currents (Isc) under basal conditions, forskolin (10^{-5} M) stimulation, bumetanide (10^{-4} M) inhibition, glucose (5mM, apical) addition, and phloridzin (10^{-4} M) inhibition. Note the absence of increased phloridzin response in older CF rabbit in stable health conditions, as compared to the one shown in Figure 1D for younger rabbits. N = 3-4/genotype *$p < 0.05$ different than WT. Unpaired, two-tailed t-test with Welch's correction for unequal variance.

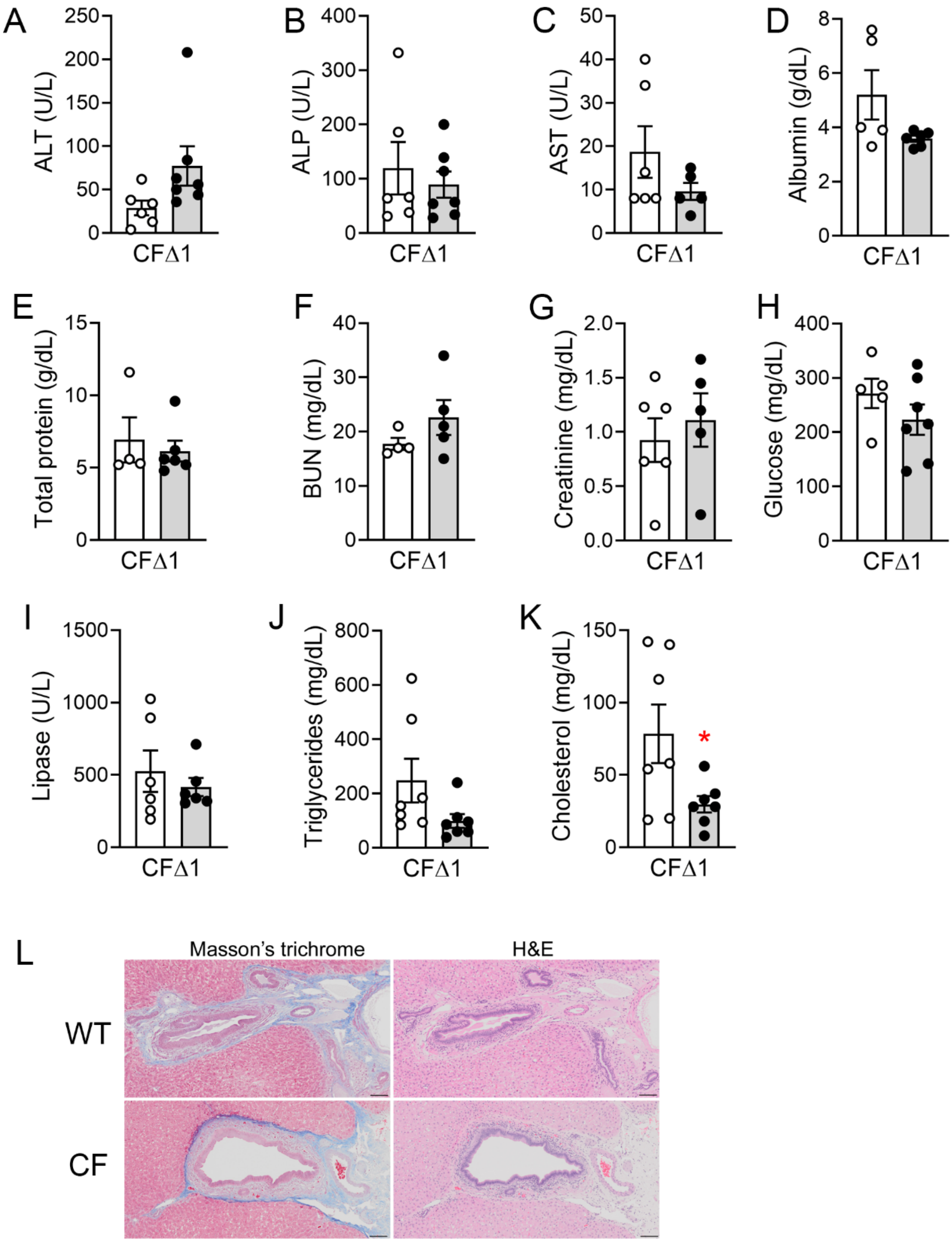

Supplemental Figure 4. **Additional characterization of WT and CF rabbit blood metabolic panel and liver histopathology. A-K**) Blood Chemistry panel for liver, pancreatic, and renal parameters in ~1 year-old WT and Δ1 CF rabbits raised at UNC (n=4-7/genotype). Abbreviations: Alanine aminotransferase (ALT), alkaline phosphatase (ACP), aspartate aminotransferase (AST), gamma-glutamyl transpeptidase (GGT), lactic acid dehydrogenase (LDH), blood urea nitrogen (BUN). * $p < 0.05$ CF vs. WT, unpaired, two-tailed t-test. **L**) Representative histological micrographs of WT and CF rabbit liver, stained for collagen (Masson's trichrome) or H&E, centered on main portal triads. Scale bar = 0.1mm. Representative micrographs from n=3 CF and control rabbits.

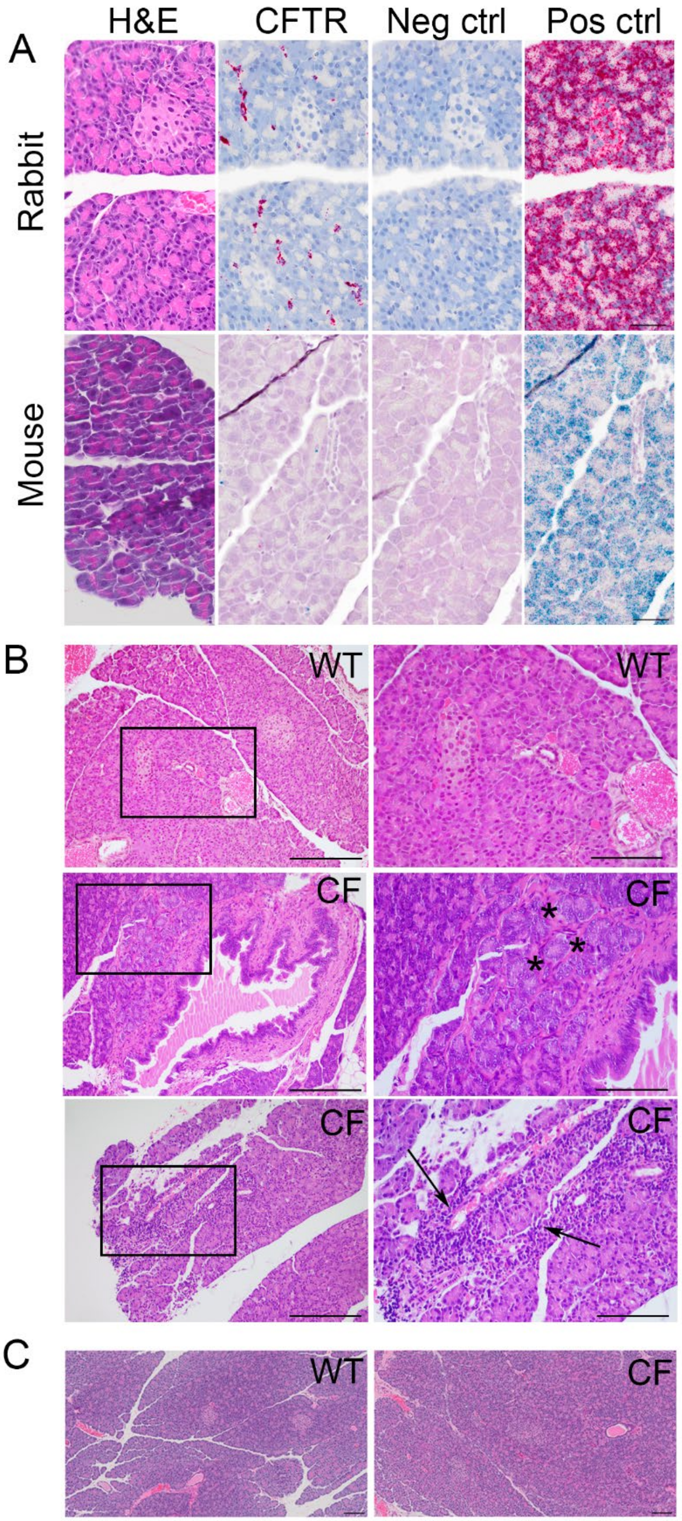

Supplemental Figure 5. **CFTR mRNA expression in WT rabbit pancreas and pancreas histopathology. A**) Complete H&E and CFTR RNAscope® panel (including CFTR, Negative, and Positive mRNA probes) for WT rabbit (red chromogen) and WT mouse (teal chromogen) pancreas. Scale bar = 20µm. Representative micrographs from n=2 rabbits and 2 mice. **B**) Representative histological micrographs of the focal lesion observed in the pancreas of 3 out of 5 Δ9 CF rabbits at UM. Region highlighted by boxes in the left panels (scale bar 0.2mm) are presented at higher magnification in the right panels (scale bar 0.1mm). Asterisks indicate areas of exocrine gland distension and fibrosis. Arrows indicate inflammatory infiltration. H&E stain. **C**) Representative histological micrographs of CF and WT rabbit pancreas from the UNC Δ1 cohort, where no pathological changes were observed. Scale bar = 0.1mm. H&E stain. Representative micrographs from n=6-7 CF and control rabbits.

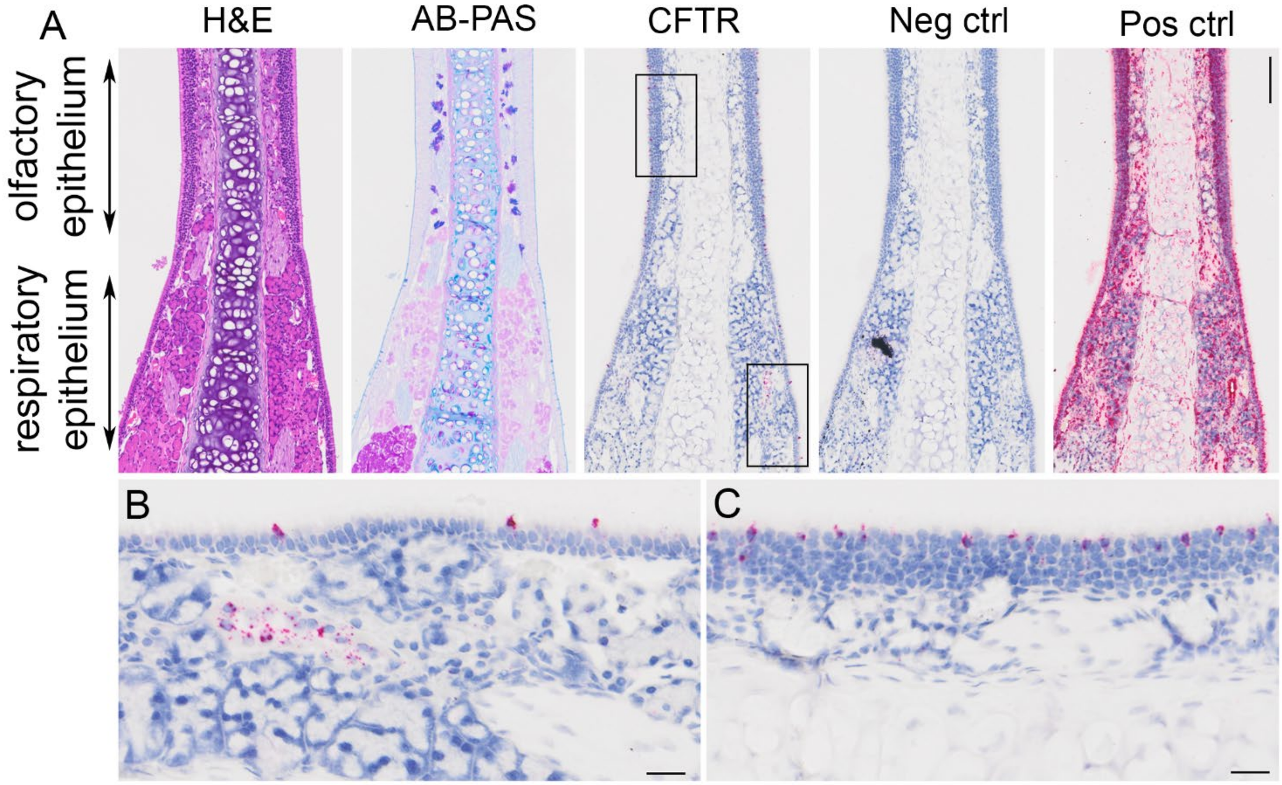

Supplemental Figure 6. **Characterization of CFTR expression in the murine nasal cavity. A**) Complete H&E, AB-PAS, and CFTR RNAscope® panel (including CFTR, Negative, and Positive mRNA probes, red chromogen) for respiratory and olfactory epithelia in the nasal cavity of WT mice. Scale bar = 0.1 mm. **B-C**) High power magnification of mouse respiratory (**B)** and olfactory (**C**) nasal epithelium regions of interest highlighted with boxes in the low-magnification images above. Note the clustered distribution of CFTR in specific cell types in both of these regions. Scale bar= 20μm. Representative micrographs from n=2 mice.

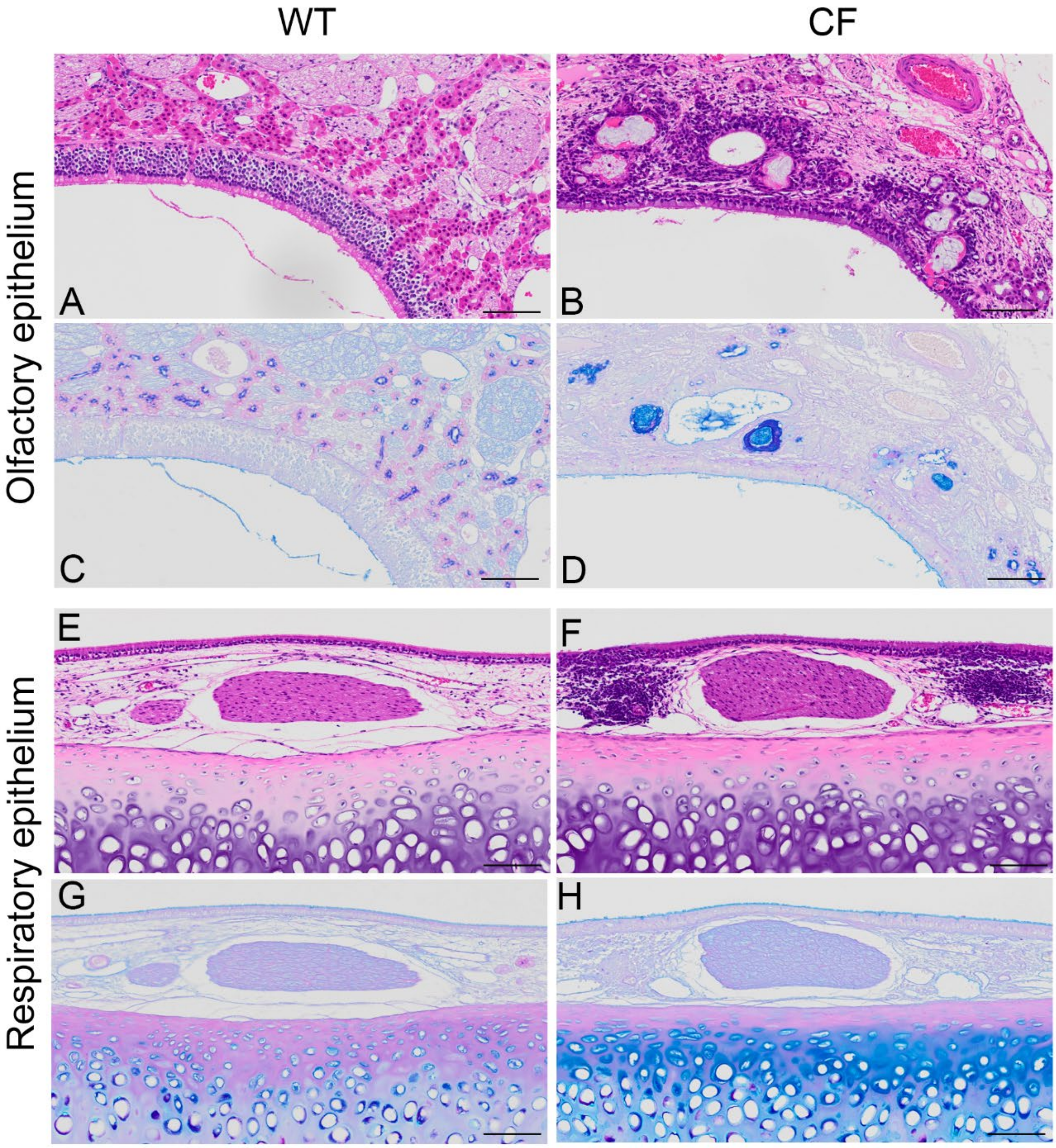

Supplemental Figure 7. **Nose pathology in CF rabbits**. Additional evidence of inflammatory remodeling in the olfactory (**A-D**) and respiratory (**E-H**) mucosa of CF (**B, D, F, H**) vs. WT (**A, C, E, G**) rabbit nose. Scale bar = 0.1mm

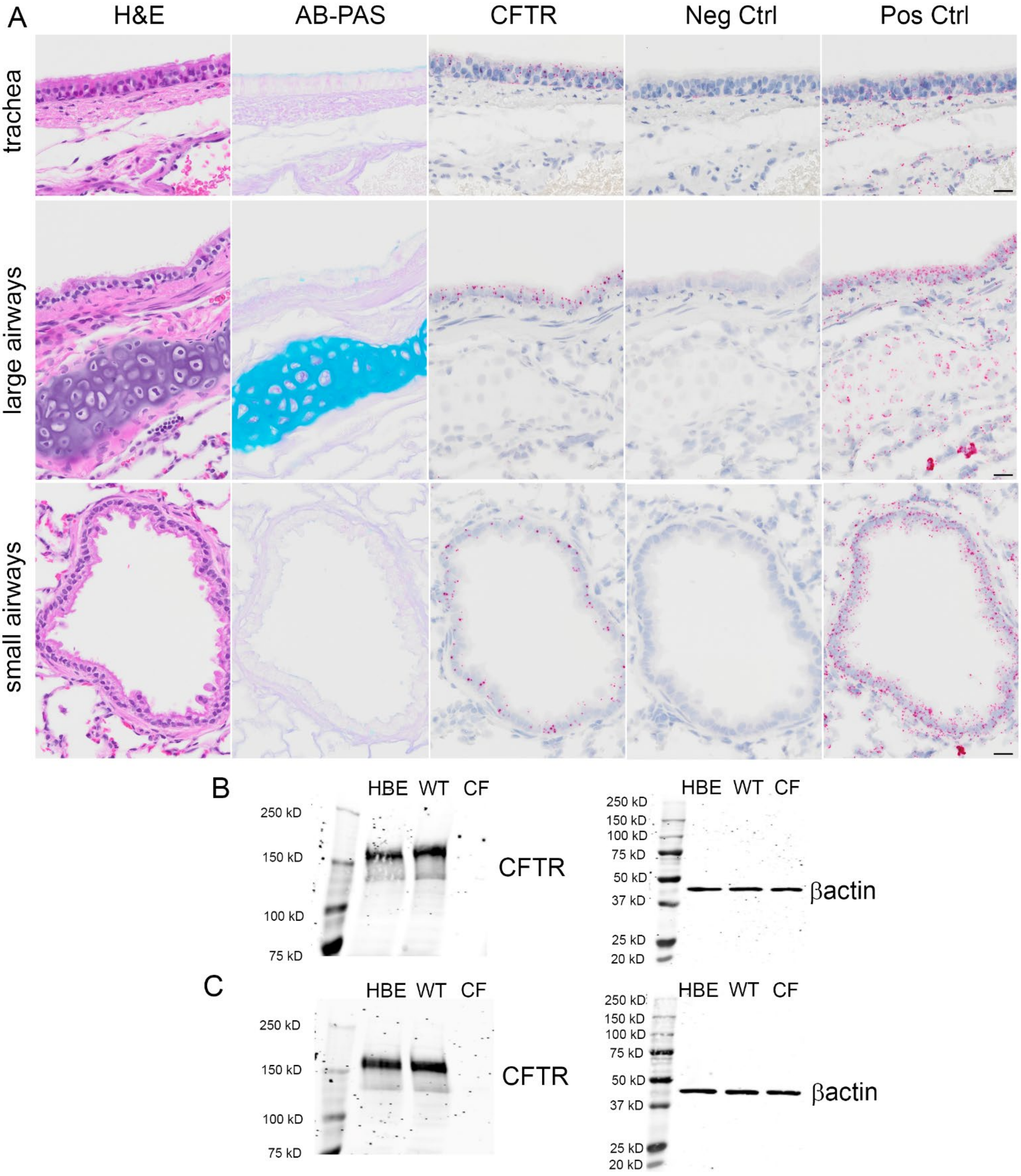

Supplemental Figure 8. **CFTR mRNA expression in WT rabbit lower airways. A)** Complete H&E, AB-PAS, and CFTR RNAscope® panel (including CFTR, Negative, and Positive mRNA probes, red chromogen) for the lower airways of WT rabbits (~ 3 months-old). Scale bar 20μm. Representative micrographs from n=2 rabbits. **B-C**) Uncropped western blots for CFTR and βactin on HBE and tracheal tissue lysates from WT and CF rabbit line Δ1 (**B,** shown in Figure 6B) and line Δ9 (**C**) with labels for protein markers' molecular weight.

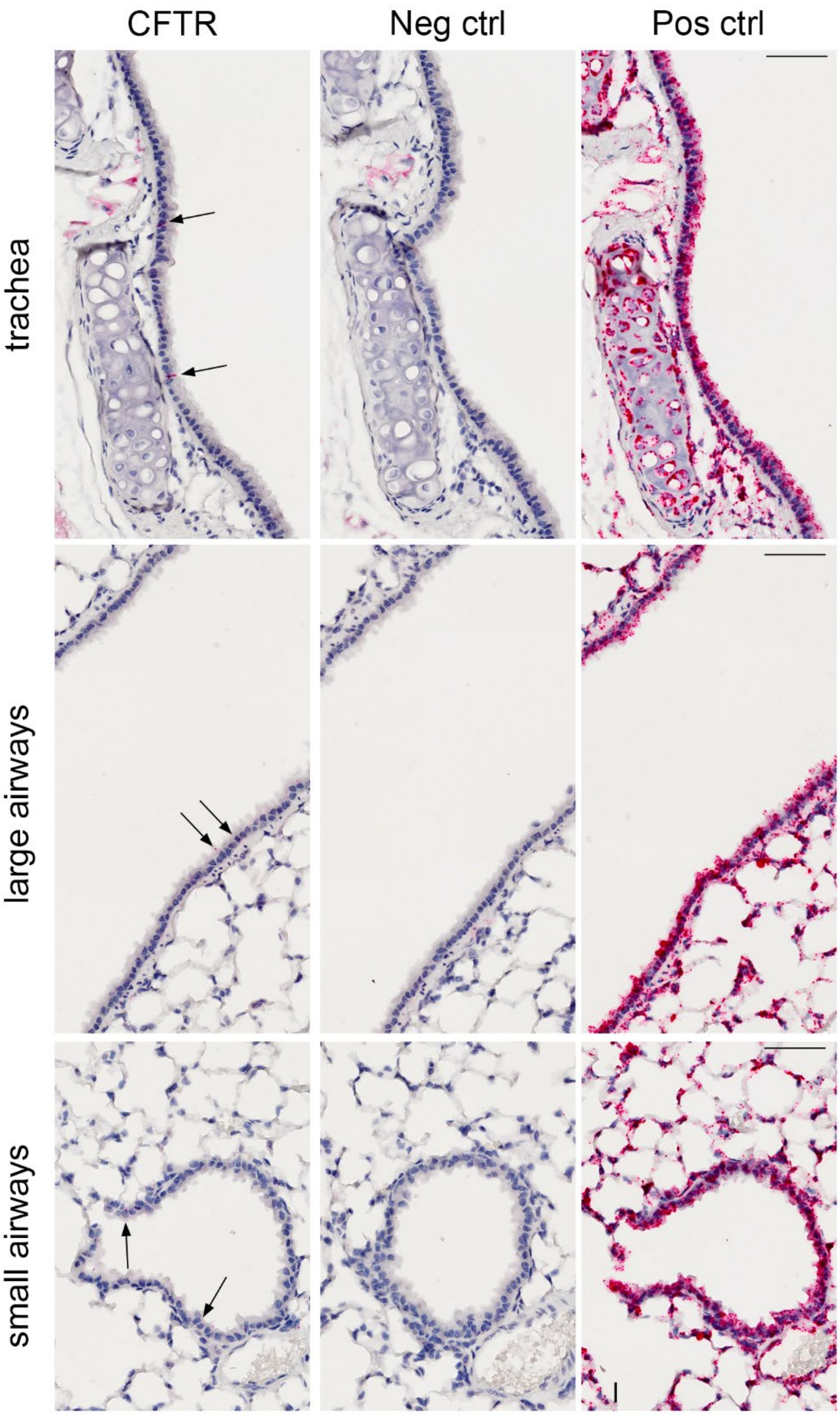

Supplemental Figure 9. **CFTR mRNA expression in WT mouse lower airways.** Complete CFTR RNAscope® panel (including CFTR, Negative, and Positive mRNA probes, red chromogen) for the trachea, large and small airways of WT mice (PND56). Arrows indicate CFTR-positive cells. Scale bar 20µm. Representative micrographs from n=2 mice.

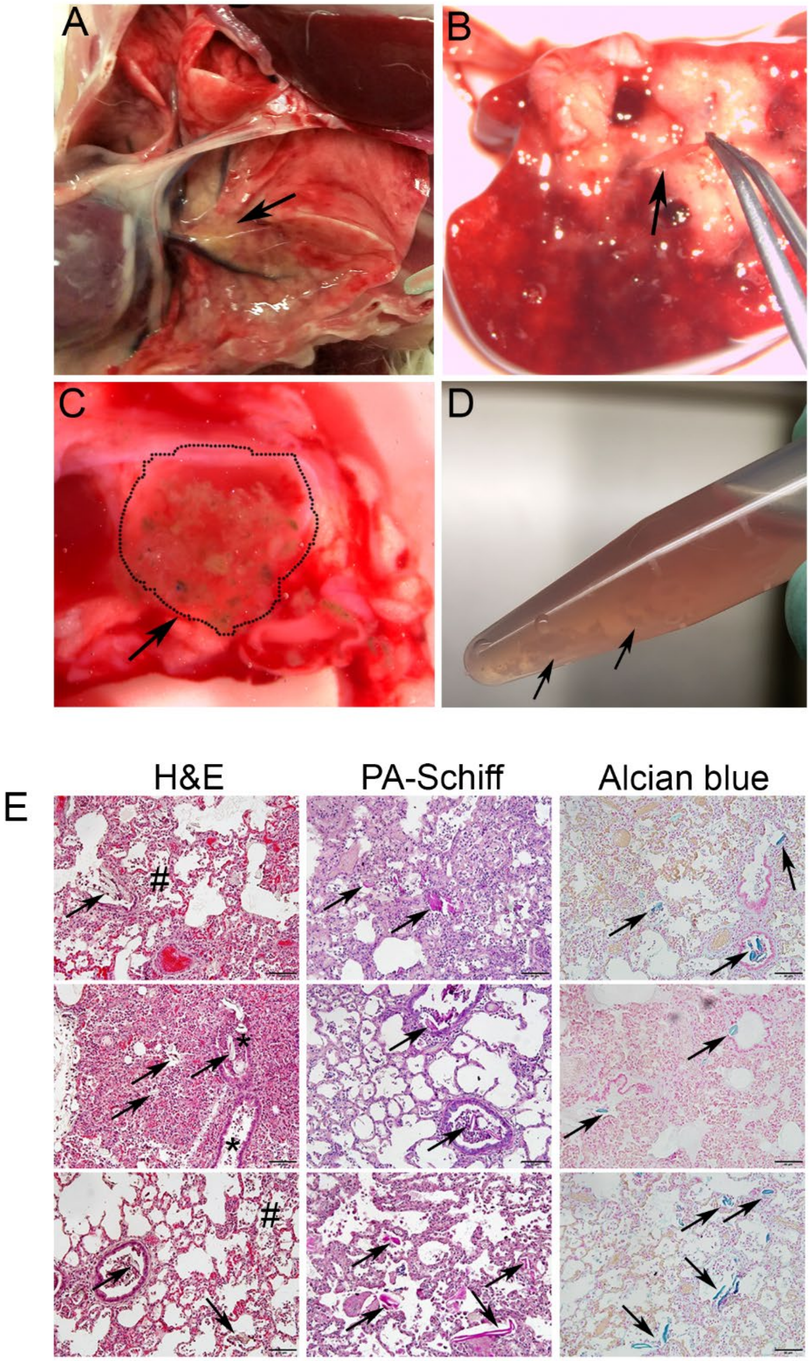

Supplemental Figure 10. Evidence of unresolved aspiration in a subset of CF rabbits after oral Golytely administration starting at PND6.
A) Gross appearance of lungs from a subset of CF rabbit syringe-fed with Golytely starting at PND6. Note the consolidated/yellowish appearance of the lung parenchyma (arrow). **B-D**) Strings of mucus-like material could be pulled out of the airways (**B**, arrow), occasionally contained particulate yellow/green material (**C**, dotted line highlights the main stem bronchus circumference), and could be isolated by BAL (**D**, arrows. The presence of blood in BAL suggests lung injury). **E**) Representative micrographs of lung sections stained with H&E, Periodic-Acid Shiff and Alcian blue, illustrating the presence of particles deposited in the airway and alveolar spaces (arrows), along with airway (*) and parenchymal (#) inflammatory cell infiltration in the lungs of a subset of CF rabbits undergoing oral Golytely administration starting at PND6.

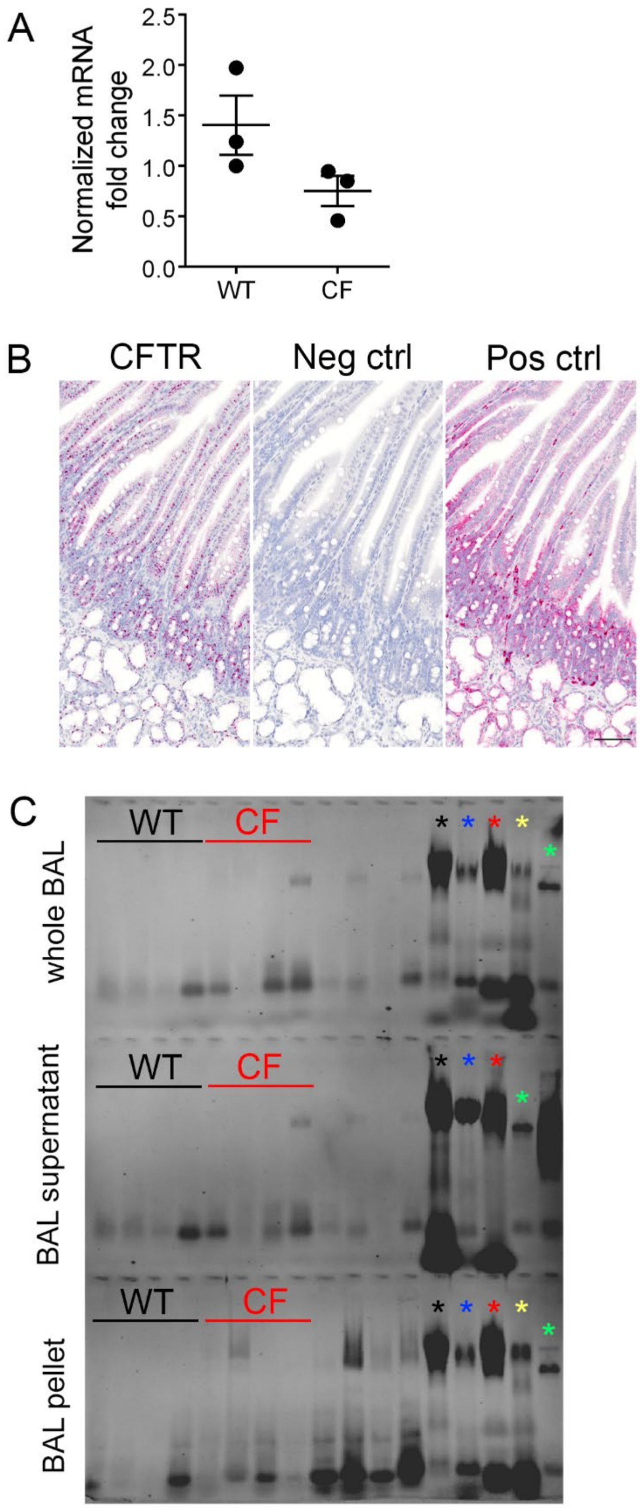

Supplemental Figure 11. CFTR mRNA expression in CF rabbit tissues and rabbit MUC5B agarose western blot. A) CFTR mRNA expression in the lungs of WT vs. CF rabbits (line Δ1) as assessed by qPCR. Unpaired, two-tailed t-test p=0.12. **B**) Complete CFTR RNAscope® panel (including CFTR, Negative, and Positive mRNA probes, red chromogen) for CF rabbit jejunum, indicating that CFTR mRNA is still present and detectable in specimens harvested from CF Δ1 rabbits. Scale bar = 0.1mm. Pancreas, proximal colon, trachea, and lungs from the same CF rabbit were also probed, yielding similar results. **C**) Validation of a goat polyclonal antibody to probe for MUC5B in rabbit bronchoalveolar lavage samples. WT (lanes 1-4) and CF (lanes 4-8) samples (unfractionated/whole BAL, BAL supernatant and pellet fractions obtained after low speed centrifugation) were run under reduced conditions and probed with a polyclonal goat antibody raised against an immunogenic peptide of mouse Muc5b (See Methods). Lane 9-12 contain samples not discussed in the current manuscript. Asterisks indicate samples of concentrated mucus harvested from the nasopharyngeal region of WT (black and blue asterisks) or CF (red and yellow asterisks) rabbits, which was used as a positive control, given the extreme dilution of the BAL samples. The lanes marked with green asterisks contain BAL sample harvested from WT mice. This control was used to verify high molecular weight migration of the putative rabbit MUC5B band, and to distinguish it from aspecific low-molecular bands. Rabbit MUC5B appears to migrate at a slightly higher MW compared to murine MUC5B.

Supplemental Table 1. Amino acid identity between human CFTR and that of species that have been genetically modified to model CF

	Human	Mouse	Rat	Pig	Ferret	Sheep	Rabbit
NCBI Accession #	*P13569.3*	*P26361.2*	*P34158.3*	*Q6PQZ2.1*	*Q07E16.1*	*NP_001009781.1*	*Q00554.4*
Human	-						
Mouse	78%	-					
Rat	78%	92%	-				
Pig	92%	79%	78%	-			
Ferret	92%	79%	78%	92%	-		
Sheep	91%	77%	77%	93%	90%	-	
Rabbit	92%	80%	79%	91%	91%	89%	-

Supplemental Table 2. Primers used for off-target analysis of sgRNA-02

Number	Primer Name	Sequence(5' to 3')
1	CF-offtarget1-F(385)	ACAGGCAGAAGAAAGGATG
2	CF-offtarget1-R(666)	CAGGAGTGAGGCTTGAGTT
3	CF-offtarget1-Seq(418)	GAGGCACAGATTTGAGTGGG
4	CF-offtarget2-F(110)	GTATCAACCTGGTGTTTCTACTT
5	CF-offtarget2-R(869)	TGTCCCAATTCTGCTCCT
6	CF-offtarget2-Seq(151)	TGCCAACCACCTGTTTAAGACT
7	CF-offtarget3-F (144)	CTCCACGCTCGGCTATTCTG
8	CF-offtarget3-R (692)	CCTTCTGTCCGCTGGTTCATT
9	CF-offtarget3-Seq(345)	GGCTCCCTGCCCTGCTGCGTTCA
10	CF-offtarget4-F(216)	TGCTGTGGTAGGCTGAAATG
11	CF-offtarget4-R(834)	TGGGAGCTTGGAGCTAAGTG
12	CF-offtarget4-Seq(286)	GACCTTATTTGGAAACAGGGACTTGG
13	CF-offtarget5-F(422)	TAACACTACTATGAAGGCAAAC
14	CF-offtarget5-R(978)	CCTGGCATGGCAAATGA
15	CF-offtarget5-Seq (757)	GGAAGTGGAATCTGATGTGCC
16	CF-offtarget6-F(197)	CCTTTGGCTACACTTTAGACCC
17	CF-offtarget6-R(874)	GCTGCTCATTCATCCGTTCT
18	CF-offtarget6-Seq(232)	GCTGAAAGCGGGAGACAGGTCA
19	CF-offtarget7-F(198)	CTGAGTGAGGAGGGAGCA
20	CF-offtarget7-R(905)	AAGGTGGACTTGAGTATGAAC
21	CF-offtarget7-Seq(301)	GCAGCCGAGTTTCCATCCC
22	CF-offtarget8-F(429)	TGGGCATCCTTAGCAAT
23	CF-offtarget8-R(778)	GTGTCCTCCTAGAAAGACTGA
24	CF-offtarget8-Seq(678)	TTCCAGGGCGCTCCTTAT
25	CF-offtarget9-F(422)	GTGACAAGAGCCGTGGAG
26	CF-offtarget9-R(969)	CGCTAGGCGGAGGATTA
27	CF-offtarget9-Seq(678)	GCTTCTTCCAGGTCTCCCACAT
28	CF-offtarget10-F(211)	CCCACATAGAGGCAGAAATACAG
29	CF-offtarget10-R(880)	AGCAATCTCATCCATCGGTTC
30	CF-offtarget10-Seq(289)	TGACCTTGACCTCCCGCTCCG
31	CF-offtarget11-F(80)	CACTTCTATTCCCACTGTTAC
32	CF-offtarget11-R(840)	GCACCTGGCTCCTGACT
33	CF-offtarget11-Seq(303)	CCCAGTTATCCTGAACAAACATC
34	CF-offtarget12-F(95)	ATGGCACAGTGGGTTCAG
35	CF-offtarget12-R(830)	TCTCCCATCTGCTTCTTCA
36	CF-offtarget12-Seq(162)	GCTGCTTCATTGCTAATGTGGG
37	CF-offtarget13-F(210)	CTGAATGTCGCACTTTGG
38	CF-offtarget13-R(782)	CCGCCTCACTTTCTGTT
39	CF-offtarget13-Seq(715)	GCAAGGGTGTCTTCCTTACTG
40	CF-offtarget14-F(252)	GCTTTGGCTCCTTTGTG
41	CF-offtarget14-R(668)	CGAGGCTTCCTCTTCTG
42	CF-offtarget14-Seq(343)	GGTATGGCGCTATCTTGTTT
43	CF-offtarget15-F(170)	AGATTCCGTTCCCTGTTC
44	CF-offtarget15-R(920)	ACTGCGTATTTACGTCTTATGT
45	CF-offtarget15-Seq(319)	GCCAACCGTGGGAAACC
46	CF-offtarget16-F(3)	ATCTCACTCCGAGGTCCAG
47	CF-offtarget16-R(753)	TCATCCATTCAAAGGCACA
48	CF-offtarget16-Seq(307)	AGTTCTGGGACGGAGGGAGTT
49	CF-offtarget17-F(429)	CAAGACGCAGACGGAACGA
50	CF-offtarget17-R(731)	AGCTCAGGCCAGGTGAGGAT
51	CF-offtarget17-Seq(606)	TGGCCGTCATCTCAGAATCCTACCTC
52	CF-offtarget18-F(309)	TCTCCTATGTGGGTGCTA
53	CF-offtarget18-R(809)	CCCTAACTGCCTCTTCA
54	CF-offtarget18-Seq(387)	GTGGAGCCTCTGGGACTTG
55	CF-offtarget19-F(419)	GATGTGATTAGATGCCAAGA
56	CF-offtarget19-R(784)	ACTGCTGACACCCGTTT
57	CF-offtarget19-Seq(664)	GTACATTATTAGCTTCCCAGCA
58	CF-offtarget20-F(150):	TCTTATCCAGCTTACATCAAG
59	CF-offtarget20-R(685)	ATTCTGTCAGCCGAGGG
60	CF-offtarget20-Seq(342)	GAGATAAGATTCAGCTCACAGCCTAC

Supplemental Table 3. Sequence analysis of predicted off-target loci of sgRNA-02. The genomic DNA from 3 CFTR KO founder rabbits were analyzed. Genomic DNA sequences with 5-7 mismatches near 5' end to the sgRNAs were computationally identified in the rabbit reference genome, and analyzed by Sanger sequencing. No off-target events were detected in these founder animals. PAM sequence is shown in blue, mismatches in the protospacer sequence are highlighted in red. #mm: number of mismatches.

No.	genomic location	locus details	Sequence	#mm	I
	sgRNA	exon CFTR	GGAGAGTTGGAGCCTTCAGAGGG		
1	Chr 1:112376551-112376557	Intron PDGFD	TGCCCTCAGGAGCCTTCAGAAGG	7	r
2	Chr 12:23852170-23852176	Intergenic	CTAGCCGTGGAGCCTTCAGAAGG	5	r
3	Chr 12:31324798-31324804	Intergenic	CCTGGCCGGGAGCCTTCAGAGGG	7	r
4	Chr 13:13290345-13290351	Intergenic	CCTCCCCTGGAGCCTTCAGATGG	7	r
5	Chr 13:34092296-34092302	Intergenic	AATCTCTTGGAGCCTTCAGATGG	6	r
6	Chr 13:36533825-36533831	Intron LOC103350042	CTTGCAGGGGAGCCTTCAGAGGG	7	r
7	Chr 14:89379526-89379532	Intron ATP13A4	TTGGAGATGGAGCCTTCAGAGGG	5	r
8	Chr 15:38720980-38720986	Intergenic	GTAGGACTGGAGCCTTCAGAGGG	4	r
9	Chr 15:107380356-107380362	Intergenic	TTTCCCTTGGAGCCTTCAGATGG	6	r
10	Chr 16:64652169-64652175	Intergenic	TCCGGAAGGGAGCCTTCAGACGG	7	r
11	Chr 19:18221198-18221204	Intron EFCAB5	GTGGCATGGGAGCCTTCAGAAGG	5	r
12	Chr 19:38629041-38629047	Intron LOC100351350	CTCCCTCTGGAGCCTTCAGATGG	7	r
13	Chr 19:54725909-54725915	Intergenic	GTGACATGGGAGCCTTCAGAAGG	6	r
14	Chr 2:71843643-71843649	Intergenic	CTATCCCTGGAGCCTTCAGATGG	6	r
15	Chr 2:74167517-74167523	Intergenic	ATGAGGGTGGAGCCTTCAGATGG	6	r
16	Chr 2:139877745-139877751	Intron EPAS1	AATGACCAGGAGCCTTCAGATGG	6	r
17	Chr 2:159124140-159124146	Intron DPYSL5	GTGCATGCGGAGCCTTCAGAAGG	6	r
18	Chr 2:167420450-167420456	Intergenic	AAAGTATGGGAGCCTTCAGAAGG	5	r
19	Chr 20:17982202-17982208	Intergenic	TCATCCCTGGAGCCTTCAGAGGG	6	r
20	Chr 3:49773422-49773428	Intron WWC1	TTCTGTGTGGAGCCTTCAGAGGG	7	r

Supplemental Table 4. Summary of main phenotypes for CF rabbits observed across study sites, i.e., UNC, UM, and WSU. n/a = not available or only limited data available

		CF ∆1 line	CF ∆9 line	CF +1 line
Mutation type		premature stop codon after amino acid 477	deletion of amino acids P477, S478, E479	premature stop codon after amino acid 480
Lifespan (median)	UNC	44 d with Golytely only, >80 d with Golytely and mucokinetic agents	n/a	n/a
	UM	n/a	45 d with Golytely only	43 d with Golytely only
	WSU	44 d with Golytely only	n/a	n/a
Male infertility	UNC	Yes	n/a	n/a
	UM	Yes	Yes	Yes
	WSU	Yes	Yes	Yes
Retarded growth	UNC	Yes	n/a	n/a
	UM	Yes	Yes	Yes
	WSU	Yes	Yes	Yes
GI obstruction	UNC	Yes	n/a	n/a
	UM	Yes	Yes	Yes
	WSU	Yes	Yes	Yes
Spontaneous upper airway disease (mucus obstruction and inflammation)	UNC	Yes, late onset (> 6 months old)	n/a	n/a
	UM	n/a	n/a	n/a
	WSU	n/a	n/a	n/a
Spontaneous lower airway disease (mucus obstruction and inflammation)	UNC	No, traces in CF rabbits > 1 year old	n/a	n/a
	UM	n/a	n/a	n/a
	WSU	n/a	n/a	n/a
